# Gene Expression Profiles Link Respiratory Viral Infection, Platelet Response to Aspirin, and Acute Myocardial Infarction

**DOI:** 10.1371/journal.pone.0132259

**Published:** 2015-07-20

**Authors:** Jason J. Rose, Deepak Voora, Derek D. Cyr, Joseph E. Lucas, Aimee K. Zaas, Christopher W. Woods, L. Kristin Newby, William E. Kraus, Geoffrey S. Ginsburg

**Affiliations:** 1 Center for Applied Genomics and Precision Medicine, Duke University Medical Center, Durham, North Carolina, United States of America; 2 Department of Medicine, Duke University Medical Center, Durham, North Carolina, United States of America; 3 Division of Pulmonary, Allergy and Critical Care Medicine, Department of Medicine, University of Pittsburgh, Pittsburgh, Pennsylvania, United States of America; Medical Faculty, Ludwig Maximilians University Munich, GERMANY

## Abstract

**Background:**

Influenza infection is associated with myocardial infarction (MI), suggesting that respiratory viral infection may induce biologic pathways that contribute to MI. We tested the hypotheses that 1) a validated blood gene expression signature of respiratory viral infection (viral GES) was associated with MI and 2) respiratory viral exposure changes levels of a validated platelet gene expression signature (platelet GES) of platelet function in response to aspirin that is associated with MI.

**Methods:**

A previously defined viral GES was projected into blood RNA data from 594 patients undergoing elective cardiac catheterization and used to classify patients as having evidence of viral infection or not and tested for association with acute MI using logistic regression. A previously defined platelet GES was projected into blood RNA data from 81 healthy subjects before and after exposure to four respiratory viruses: Respiratory Syncytial Virus (RSV) (n=20), Human Rhinovirus (HRV) (n=20), Influenza A virus subtype H1N1 (H1N1) (n=24), Influenza A Virus subtype H3N2 (H3N2) (n=17). We tested for the change in platelet GES with viral exposure using linear mixed-effects regression and by symptom status.

**Results:**

In the catheterization cohort, 32 patients had evidence of viral infection based upon the viral GES, of which 25% (8/32) had MI versus 12.2% (69/567) among those without evidence of viral infection (OR 2.3; CI [1.03-5.5], p=0.04). In the infection cohorts, only H1N1 exposure increased platelet GES over time (time course p-value = 1e-04).

**Conclusions:**

A viral GES of non-specific, respiratory viral infection was associated with acute MI; 18% of the top 49 genes in the viral GES are involved with hemostasis and/or platelet aggregation. Separately, H1N1 exposure, but not exposure to other respiratory viruses, increased a platelet GES previously shown to be associated with MI. Together, these results highlight specific genes and pathways that link viral infection, platelet activation, and MI especially in the case of H1N1 influenza infection.

## Introduction

Influenza viral infections are associated with an increased risk of myocardial infarction (MI), in part, due to an association with platelet activation [1][2][3][4]. The H1N1 strain of influenza has been associated with an acute MI in one case report of a young patient without coronary artery disease [5]. Similar associations may exist for other influenza strains (e.g., H3N2) and other respiratory viruses, but are not as frequently reported [6][7][8]. These associations suggest that viral infection (or exposure) may induce biologic pathways that contribute to MI.

Microarray analysis is used as a genome-wide assessment of gene expression. Compared with individual gene expression, the use of gene-expression “signatures”, or groups of genes with similar expression patterns, can be used to represent the activities of certain biological pathways, some of which have yet to be functionally defined. We have recently identified a peripheral blood gene expression signature (GES) of viral infection that can identify individuals with symptomatic, respiratory viral infection with >95% accuracy and classify viral versus non-viral acute respiratory infection with >93% accuracy [9][10][11][12] (viral GES). In a separate study, we used peripheral blood gene expression profiling to identify and to validate a GES correlative of platelet function in response to aspirin (platelet GES). This signature was primarily made up of platelet genes and was predictive of death or MI in patients with cardiovascular disease [13]. Because the platelet GES was not correlative of platelet function in the absence of aspirin, the platelet GES can be thought of as being reflective of aspirin’s effect on platelet function; lower levels are indicative of a greater aspirin effect on platelets and lower risk for death/MI.

Here we test the hypotheses that 1) biological pathways that change in response to viral infection are associated with MI and 2) viral exposure and/or viral infection are associated with an aspirin-responsive platelet pathway that is associated with MI.

## Materials and Methods

### Overview

We used existing microarray data from two prior studies: 1) gene expression data from patients at the time of cardiac catheterization (Database of Genotypes and Phenotypes accession numbers: phs000548.v1.p1 and phs000551.v1.p1) [9] to test Hypothesis #1 and 2) serial gene expression from prospective cohorts of healthy volunteers exposed to four different respiratory viruses (Gene Expression Omnibus database accession numbers GSE17156 and GSE52428) [11] to test Hypothesis #2.

### CATHGEN Cohort

From 2004–2010, the Catheterization Genetics (CATHGEN, http://cathgen.duhs.duke.edu) biorepository banked whole blood RNA in PAXgene blood tubes from Duke University Medical Center patients at the time of cardiac catheterization [10]. Baseline medical history, medication usage, clinical and procedural characteristics are available through the Duke Databank for Cardiovascular Disease. Two previously defined cohorts of CATHGEN participants with available microarray data were combined for analysis, yielding 594 unique patients [13][14]. The primary outcome was MI in the CATHGEN cohort. All potential MI cases were verified by chart and laboratory data review through chart review by an unblinded internist. MI occurring after the initial catheterization was excluded. The following criteria were used to determine MI: 1) MI was the indication for catheterization, 2) myocardial infarction listed in the discharge summary or admission history and physical, and 3) elevated of CK-MB or troponin above the local upper limit of normal. Eighty potential MI cases were identified and selected for chart review, of which 3 were excluded (not considered MI cases), leaving 77 confirmed MI cases. The remaining 517 patients served as non-MI controls. Because ST-segment elevation myocardial infarction (STEMI) is a more homogeneous clinical condition that is less likely to be confused with non-thrombotic causes of MI (such as demand ischemia or myocarditis), we further classified MI as STEMI if the following criteria were met: 1) meeting criteria for MI and 2) STEMI diagnosis listed in the discharge summary and/or admission history. Of the 77 MI cases (which included non-ST segment elevation myocardial infarction (NSTEMI) and STEMI), 12 (15.6%) were determined to be STEMI based on chart review and the remaining classified as NSTEMI.

### Viral Infection Cohorts

**Fig 1:**
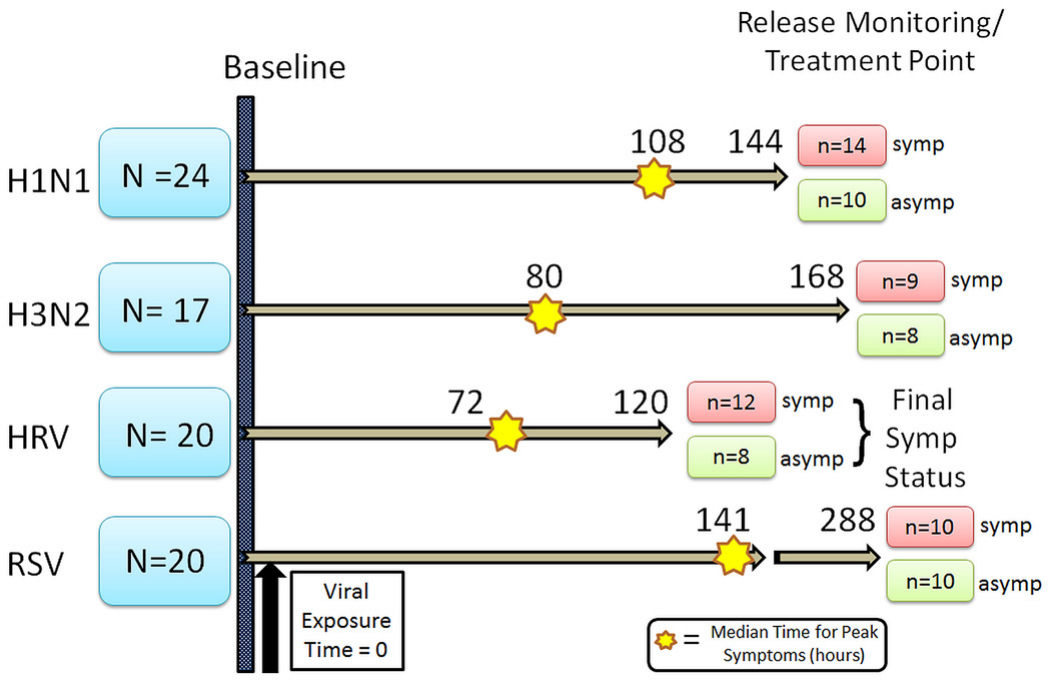
Design of viral exposure of infection cohort patients. Four cohorts of healthy volunteers were exposed to different viruses (H1N1—Influenza A (A/Brisbane/59/2007); H3N2—Influenza A A/Wisconsin/67/2005 (H3N2); HRV—Human rhinovirus; RSV—Respiratory Syncytial Virus). Blood RNA data were collected at baseline and at additional timepoints following viral exposure to assess for changes in the platelet gene expression signature (platelet GES) (S1 Table) Median time post exposure for peak symptom of each respective virus is shown (in hours). The point of treatment with Tamiflu (H1N1 and H3N2) or release from quarantine is shown as end of arrow (in hours). The final numbers of symptomatic (symp) or a symptomatic (asymp) status of each cohort is also shown.

Four separate healthy volunteer cohorts were each exposed to a different viral strain, and then monitored for symptoms, and RNA was ascertained at different time points as previously described [9][11] (Fig 1). In general, “healthy” was defined as absence of any significant acute or chronic, uncontrolled medical or psychiatric illness, that in was associated with increased risk of complications of respiratory viral illness (subjects with uncomplicated chronic diagnoses stable and treated for three [3] months, e.g., mild hypertension well-controlled with medication, were enrolled—provided the condition and its therapy are not known to be associated with an immunocompromised state or increased risk of complications of respiratory viral illness). Although certain concomitant medications were allowed, none were on aspirin at the time of viral challenge. Subjects were classified as symptomatic vs. asymptomatic based on the Modified Jackson Score [15][16][17].

### Definition of Viral GES

We have previously used two approaches, factor modeling and factor model projection [13][18] to reduce the dimensionality of microarray data and to generate gene expression signatures. Briefly when applied to a microarray datasets, a factor model generates a series of “factors”, which are sets of coexpressed transcripts representative of a potentially unknown biological pathways. Each sample can be assigned a “factor score”, which represents the aggregate expression of each of the transcripts within a factor. The factor scores can then be used for association testing with phenotypes of interest. In order to estimate factor scores in new datasets, factor model projection is used.

In the infection cohorts, we previously identified a factor that discriminated symptomatic (infected) subjects (HRV, RSV or influenza A) from asymptomatic (uninfected) individuals. However, because the normalization procedures in the infection cohorts differed from that in the CATHGEN cohort, we were unable to directly project the factors from the infection cohorts onto the CATHGEN cohort microarray data. To use the same normalization procedure on both the infection and CATHGEN cohorts, we re-derived the infection cohort factors using the same methodology, and projected this re-derived factor model on the CATHGEN datasets. Using this approach we were able to derive a factor based on the RNA data collected at baseline, 72-77h, and 93-96h after exposure that, we have previously shown, clearly discriminated those with/without viral infection (p-value = 3.9e-17 and FDR q-value = 5.28e-16) [9]. This factor included 957 genes, including 38 of the top 40 genes used for factor loading for the RSV, HRV, Influenza, Panviral cohorts in Zaas *et al* [9]. Therefore, this re-derived factor recapitulates the original Zaas *et al* signature with respect to gene membership as well as ability to discriminate between symptomatic and asymptomatic viral infection. This factor is defined as the “viral GES” used in the infection and CATHGEN cohorts for all statistical analyses (Table 1).

**Table 1:** List of 72 Genes in Platelet GES and top 49 genes in the Viral GES.

Platelet GES				Viral GES		
FSTL1	CPNE5	TPM1	CDC14B	IFI35	COX4I1	STAT1
CTTN	CLEC1B	MGLL	C6ORF79	MAK8	RPl12	HNRNPC
CTDSPL	SELP	CLU	TTC7B	FAM102A	HNRNPU	CALU
TREML1	IGF2BP3	THBS1	ARHGAP6	Elf1	EIF3A	CTNNA1
SPARC	SH3BGRL2	MYL9	PARVB	CD3D	CLTC	ZYX
ITGA2B	PROS1	PF4	TUBB1	IFIH1	WARS	PSME1
CMTM5	ALOX12	GP1BB	GNG11	ELF4	SOD1	RPS15
SLC24A3	JAM3	TGFB1I1	PRSS1	STAT1	GLUL	RPL29
MPL	LRRC32	PCSK6	PRKAR2B	GAS6	S100A11	PRDX6
CLU	ITGB3	CALD1	MFAP3L	RPL18	DNAJB1	H2AFZ
TMEM64	PPBP	GUCY1B3	ENDOD1	RpL19	SPARC	CNOT1
BEND2	RAB27B	PDE5A	FRMD3	RPS11	PPP2R1A	RNF114
MYLK	ELOVL7	PBX1	CLEC4D	RPl10a	NPC2	PSAP
C12ORF39	RHOBTB1	MMD	SDPR	C22ORF28	ACADVL	
PCGF5	HIST1H3H	PF4V1	HIST1H2AG	HSP90AB1	RPL13A	
RAB4A	HIST1H2BG	LGALSL	ARHGAP18	SNX3	RPL10	
FSTL1	CPNE5	TPM1	CDC14B	USP22	ACTR2	
CTTN	CLEC1B	MGLL	C6ORF79	PTP4A1	GNB1	

### Viral GES “cut-off”

**Fig 2:**
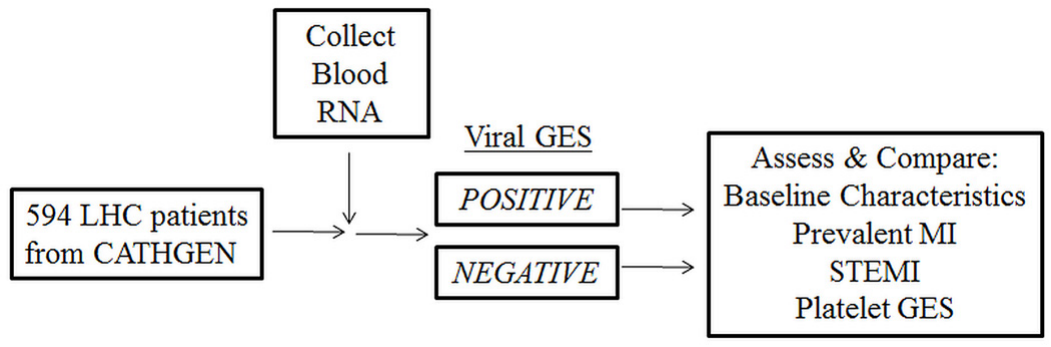
Experimental Design for CATHGEN cohort. Viral GES is projected on the 594 patients from CATHGEN, then separated into positive or negative viral GES based on a previously defined cutoff (see Methods). Baseline characteristics and MI status were compared between groups. LHC—Left Heart Catheterization

To determine a “cut-off” value of viral GES to classify viral infection in the CATHGEN cohort we generated a receiver operating characteristics (ROC) curve using microarray data from the infection cohorts to discriminate between asymptomatic and symptomatic subjects (Fig 2). The area under the ROC curve in the infection cohorts was 0.91 and the optimal cutoff (calculated as the value that achieved the maximum sum of sensitivity and specificity) was a viral GES of ≥ 0.63. The distribution of the viral GES score was approximately the same in the CATHGEN (median viral GES score = -0.56 interquartile range (IQR) [-0.84, -0.23] vs. asymptomatic time points from viral infection cohorts (median viral GES = -0.51, IQR [-0.64, -0.18]. Therefore, we directly applied the cutoff derived in the viral infection cohorts to the CATHGEN data to classify individuals as having molecular evidence of viral infection (viral GES ≥ 0.63, “positive”) or no molecular evidence of viral infection (viral GES < 0.63, “negative”). Sensitivity analyses were performed by varying the cutoff level in the CATHGEN cohort.

### Platelet GES

The platelet GES is a factor containing 62 genes, primarily of platelet origin, that was validated in two independent cohorts as a set of co-expressed genes associated with platelet function in response to aspirin (Table 1)[13]. In the same combined CATHGEN cohort used in the current work, a higher platelet GES was associated with a higher risk of death/MI following cardiac catheterization. We projected the platelet GES into the infection cohorts test for changes following viral exposure.

## Statistical Methods

### CATHGEN Cohort

Baseline characteristics are presented as medians (25^th^, 75^th^ percentiles) for continuous variables and frequencies for categorical variables in those positive vs. negative viral GES. The chi-square test was used to identify differences in categorical variables between groups. For continuous data, a student’s t-test was used test for differences in means; a Wilcoxon rank sum test was used for significant deviations from normality. We performed logistic regression in the CATHGEN cohorts to test the association of viral GES with MI or STEMI. Results are reported as odds ratios (OR) with 95% confidence interval (CI) and p-values. Student’s t-test was used to compare mean platelet GES in those with a positive vs. negative viral GES. Pearson correlation was used to test for association between the viral GES and the platelet GES.

### Infection cohorts

We used linear mixed-effects regression with random effects for subject to model the platelet GES score over time, modeled as time + time^2^. To determine the association between platelet GES and viral exposure we compared an intercept only model vs. a model with time. To assess for homogeneity of the effects of viral exposure models with and without interactions between symptom status and time were compared. All model comparisons were performed using an ANOVA likelihood ratio test. The prediction curves included in each plot are predictions made on the fixed effects only.

All analyses were performed using SAS Enterprise Guide 4.3 (SAS Institute Inc, Cary, North Carolina, USA) or R (version 2.8.1). Results were declared significant at a two-sided p-value <0.05. As this was a hypothesis generating study, no adjustments were made for multiple testing.

### Institutional Review Board (IRB) Approval

The Influenza challenge (H1N1, H3N2) protocols were approved by the East London and City Research Ethics Committee 1 (London, UK), an independent institutional review board (WIRB: Western Institutional Review Board; Olympia, WA), the IRB of Duke University Medical Center. (Durham, NC), and the SSC-SD IRB (US Department of Defense; Washington, DC) and were conducted in accordance with the Declaration of Helsinki. The other viral challenge studies (HRV, RSV) were approved by the respective site IRB/ethics boards—WIRB (RSV) and the University of Virginia IRB (HRV). All subjects enrolled in viral challenge studies and CATHGEN were provided written informed consent per standard IRB protocol. The current analyses using the databases from these trials were approved by the IRB of Duke University Medical Center.

## Results

### Hypothesis #1: Viral GES is associated with MI in CATHGEN cohorts

Of the 594 patients in the CATHGEN cohorts, 32 (5.39%) had evidence of viral infection—as determined by the presence of a viral GES score ≥ 0.63. There were no significant differences in baseline, medication, or clinical characteristics between those with positive vs. negative viral GES scores (Table 2). We assessed for the imbalance in aspirin use at the time of cardiac catheterization and found no statistically significant difference in aspirin use between the groups.

**Table 2:** Baseline characteristics of the CATHGEN cohort. Continuous variables reported as mean values, categorical variables reported as percentages.

	Positive viral GES (n = 32)	Negative viral GES (n = 562)	p-value
Characteristic	N or Mean (% or Q1,Q3)	N or Mean (% or Q1,Q3)	
Demographics			
Age	63 (53,71)	62 (54,71)	0.59*
BMI	26.4 (25,31)	28.7 (25,33)	0.12*
Female Gender	12 (37.5%)	195 (34.7%)	0.48
AA Ethnicity	11(34.3%)	123 (21.9%)	0.13
Medical History			
HTN	27 (84.4%)	391 (69.6%)	0.075
CHF	12 (37.5%)	169 (30.1%)	0.41
FH CAD	10 (31.3%)	199 (35.4%)	0.63
DM	9 (28.1%)	188 (33.2%)	0.53
History MI	12(37.5%)	173(30.8%)	0.42
Hyperlipidemia	17(53.1%)	348 (61.9%)	0.32
Smoker	18 (56.3%)	278 (49.5%)	0.46
Medications			
ACEi	20 (66.7%)	358 (68.8%)	0.80
Beta Blocker	23 (76.7%)	361 (69.4%)	0.40
Statin	18 (60.0%)	358 (68.8%)	0.31
Aspirin	22 (73.3%)	430 (82.7%)	0.19
Clopidogrel	7 (23.3%)	200 (38.5%)	0.10
Catheterization data			
EF	52.4	56.14	0.47*
CAD Index [49]	32 (0,63)	32(0,63)	0.52*
# Diseased Vessels			0.44
0	13 (44.8%)	188 (34.6%)	
1	4 (13.8%)	100 (18.42%)	
2	6 (20.7%)	99 (18.2%)	
3	6 (20.7%)	152 (28.0%)	

^#^—Number; AA—African American; ACEi = Angiotensin converting enzyme inhibitor; BMI—body mass index in kg/m^2; CAD—coronary artery disease; HTN—hypertension; CHF—congestive heart failure; EF—Left Ventricular Ejection Fraction; F—female; DM—history of diabetes mellitus; FH CAD—family history of coronary artery disease; MI—myocardial infarction; Angiotensin Converting Enzyme Inhibitor;

* indicates non-parametric, Wilcoxon rank-sum test used to test for differences between groups due to deviations from normality.

To test the hypothesis that prior viral infection was associated with MI, we tested for the association between molecular evidence of viral infection (positive/negative viral GES) and MI in the CATHGEN cohort (Table 3). Among the 32 with a positive viral GES, 8 (25.0%) had MI vs. 69 of 562 (12.3%) in those with a negative viral GES (OR = 2.3 [95% CI = 1.03–5.51], p = 0.04). To determine the extent to which the cut-off value influenced our results, we performed a sensitivity analysis by adjusting the threshold and found similar associations with MI across a range of values. (Table 3) Because STEMI is a more homogenous and readily identified clinical condition, we next limited our analysis to those with STEMI (remaining non-STEMI cases excluded). We found that 3/32 (9.38%) of those with positive viral GES had STEMI compared to 9/562 (1.60%) in those with a negative viral GES (OR = 6.36 [95% CI = 1.63–24.74] p = 0.008).

**Table 3:** Sensitivity analysis of viral threshold on association with MI.

Threshold value	Viral GES status*	MI cases	Non-MI controls	Logistic regression p-value for association with MI	Odds Ratio	95% CI
0.70	(+)	8	23	0.03	2.5	1.01–5.6
	(-)	69	494			
0.65	(+)	8	23	0.03	2.5	1.01–5.6
	(-)	69	494			
0.63**	(+)	8	24	0.04	2.3	1.03–5.5
	(-)	69	493			
0.60	(+)	8	26	0.06	2.2	0.89–4.8
	(-)	69	497			
0.55	(+)	11	27	0.004	3.0	1.4–6.2
	(-)	66	490			

*Viral GES status was determined by a viral GES greater than or equal to (+) or less than (-) a threshold value (see Methods for more details).

**Predetermined threshold value; GES = Gene expression Signature; MI = myocardial infarction; CI = confidence interval

The CATHGEN cohort has no direct measures of platelet function. However, to further examine the extent to which the association of MI and viral infection was due to a potential platelet-related biological pathway, we compared the platelet GES in those with a positive versus negative viral GES and found no association (p = 0.70). Further, we found no correlation between the platelet GES score and viral GES (p = 0.70).

### Hypothesis #2: Platelet GES changes in response to viral exposure in infection cohorts

#### H1N1

**Fig 3:**
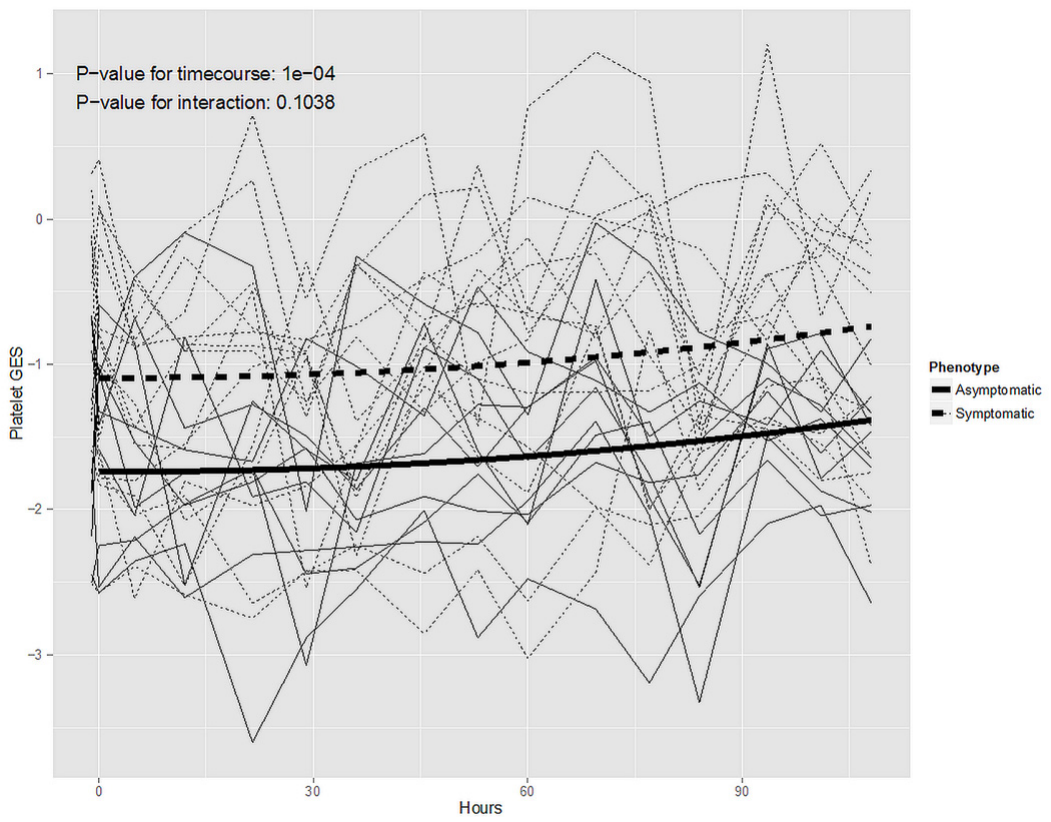
Distribution of platelet GES score by time point in the H1N1 exposure cohort. Individual platelet gene expression signature values (platelet GES, y-axis) are plotted over time (hours, x-axis) following H1N1 viral exposure and by symptom status (symptomatic/dashed thin lines; asymptomatic/solid thin lines). Prediction curves (thick lines) for the symptomatic (dashed) vs. asymptomatic (solid) subsets are plotted based on predictions made from mixed-effects regression model (see Methods). P-values represent the association between platelet GES over time and differences over time between symptomatic vs. asymptomatic subjects.

Exposure to HIN1 was associated with an increasing platelet GES over time (time course p-value = 1e-04) (Fig 3). To confirm that the individual genes represented by the platelet GES also changed in response to H1N1 exposure we tested each probe set within the signature and found that, 40 of 50 probe sets were significantly (p < 0.05) up regulated in response to H1N1 viral exposure (S1 Fig). When comparing the symptomatic vs. asymptomatic subsets, we observed that symptomatic subjects differed at baseline with no significant differences across time (Fig 3, symptom by time interaction p-value = 0.1).

### Remaining Viral Exposures

For the H3N2, RSV, and HRV cohorts we did not observe a significant association of viral exposure with platelet GES (time course p-values = 0.1, 0.2, and 0.09, respectively).

## Discussion

Despite the epidemiologic link between respiratory viral infections, including influenza, and myocardial infarction (MI), the biological pathways that underlie these associations are unknown. We hypothesized that patients with molecular evidence of viral infection would be more likely to have an MI in our cohort. We found that those patients with molecular evidence of viral infection were twice as likely (25.0% vs. 12.3%) to present with MI compared to those without. Separately, in an experimental model of viral infection we hypothesized that viral infection would affect a previously described platelet pathway of aspirin response that was associated with MI [13]. We found that H1N1 influenza exposure increased expression of genes in this aspirin response, platelet pathway. Taken, together, these findings suggest that specific biologic pathways that may mechanistically link influenza and MI.

Respiratory viral infections, in general, and influenza infection, specifically H1N1 and H3N2, are associated with an increased risk of MI [2][3][4][6][7][8][19] for up to 2 weeks after infection [20][6]. Remote infection with influenza A and B (i.e., presence of positive IgG antibodies) is also associated with acute MI [21]. These prior observations suggest that there is an initial and continued heightened risk of MI following viral infection, in particular influenza. Although H1N1 and H3N2 have both been associated with MI [7], we only found evidence that H1N1 changed platelet GES. Prior studies demonstrated that different influenza strains can produce differential effects on gene expression and cytokine induction [22][23][24]; therefore, this may not be unexpected. Alternatively, given the small numbers in each cohort we may have been underpowered to detect more subtle changes in gene expression in the non-H1N1 cohorts. Further validation will be required to confirm the H1N1 specific effects we observed in this study.

It is well known that platelets play a critical role in the development of MI. Viral infection, particularly with influenza, results in platelet hyperreactivity and activation in both human and animal models [1][25][26]. The introduction of inactivated influenza vaccine itself has been associated with platelet activation [27]. Influenza has also been shown to influence hemostasis and endothelial activation/dysfunction [28][29]. Of the 62 genes in the platelet GES up to 31 overlapped with platelet or megakaryocyte specific genes [13]. Therefore, our findings that H1N1 influenza exposure alters a platelet GES add to existing data linking influenza and platelet activation by highlighting specific platelet genes/proteins connected to this response.

**Fig 4:**
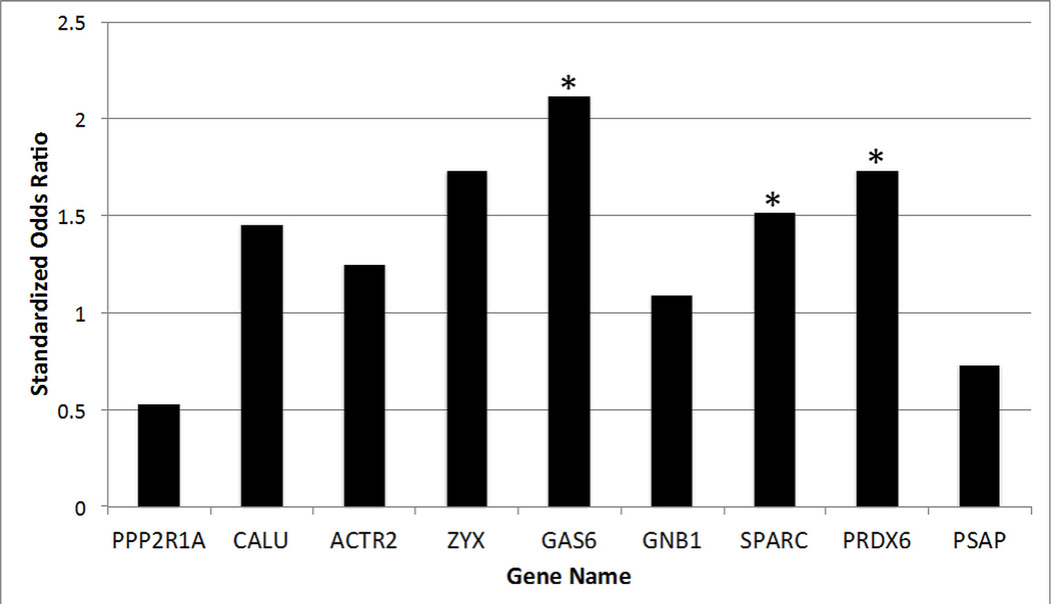
Association of selected viral gene expression signature genes with myocardial infarction. Genes from the viral gene expression signature (viral GES) were selected based on their role in platelet activation, thrombosis, and hemostasis (Table 4 and Discussion). The association between gene expression and myocardial infarction (MI) is plotted as the standardized odds ratio (y-axis) for each gene (x-axis). Higher odds ratio imply that higher gene expression is associated with higher risk of MI. * indicate genes that are significantly (p-value < 0.05) associated with MI.

The viral GES contains a large number of genes from biologically plausible gene networks involved in host viral response [30]. Of the top 49 genes, 9 (18%) are related to platelets or hemostasis (Table 4). Of particular interest is growth arrest-specific 6 (*GAS6*), which appears to link viral infection and MI. GAS6 plays a key role in platelet aggregation and vascular homeostasis [31][32] by amplifying endothelial cell activation in response to inflammatory stimuli [33]. In our study, we observed that H1N1 viral infection increases *GAS6* expression and higher *GAS6* expression is associated with a higher risk of MI (Fig 4) which is consistent prior work demonstrating that *GAS6* deficient mice are protected from thrombosis [31]. Infection then theoretically increases GAS6 which may increase thrombosis risk versus lower levels of GAS6; this could potentially be a target pathway for future study [34]. Inflammation has long been postulated to be associated with atherosclerosis, and the host inflammatory response to influenza infection may represent an alternative biological pathway by which influenza leads to MI [35]. In a case control series, patients with influenza antibodies indicative of prior influenza infection had a higher risk of MI in addition to increased levels of multiple inflammatory cytokines [36]. Therefore, the viral GES represents multiple inflammatory, coagulation, and platelet pathways that together may contribute to contribute to the development of MI after viral infection.

**Table 4:** Potential role of viral GES genes in platelet activation, thrombosis or hemostasis

*Gene*	*Description*
GNB1	Gene involved in platelet activation pathway, thrombin signaling and hemostasis [50][51]
CALU	Released by activated platelets, Expressed in atherosclerotic lesions but not normal vasculature [52][53]
PRDX6	Protective versus oxidant injury, Decreased in Influenza [54][55]
PPP2R1A	Involved in thromboxane A2 synthesis (thrombin activated platelets) [56]
PSAP	Sphingolipid metabolism, sphingolipids are involved with ischemia/reperfusion injury of the heart, found in platelets and plasma, metabolism altered in MI models [57][58]
SPARC	Glycoprotein secreted by platelets, maintains cardiac extracellular matrix after MI, down regulated in ACS patients versus controls [59][60]
ACTR2	Gene involved in hemostasis [51]
ZYX	Thrombin signaling via interaction with PAR-1 receptor, upregulated in ACS versus non-ACS patients [61][62]
GAS6	Elevated in septicemia and general inflammation, involved in vascular homeostasis and platelet aggregation, deficient mice are protected against thrombosis [31][32][33][34]

There are several, potential clinical implications if our findings are confirmed by others. Because of the strength of evidence surrounding H1N1 and H3N2 vaccination and a reduced risk of MI, vaccination is recommended in patients with coronary or atherosclerotic vascular disease [37][38][39]. Our findings provide additional, complementary data, to support current recommendations for vaccination, although vaccination itself may increase platelet activation [27]. Further, in the H1N1 infection cohort, we observed that those that did vs. did not develop symptoms after H1N1 viral exposure had similar increases in the platelet GES (Fig 3), suggesting that viral *exposure* even in the absence of *infection* may be associated with increased cardiovascular risk. Therefore in addition to vaccination programs that prevent viral infection, our findings suggest that prevention of viral exposure (i.e. though infection control programs) may additionally prevent MI in patients at risk for cardiovascular disease. Second, several studies have shown statins reduce morbidity and mortality in patients with influenza infections [40][41]. Statins have acute effects in reducing inflammation and platelet activation—two pathways represented by viral GES genes—and stabilizing atherosclerotic plaque. Therefore, there may be role for statin therapy following influenza infection for CVD prevention. Last, prior work by others links platelet activation with influenza infection [42]. Our findings link an aspirin-responsive pathway to the heightened platelet activation observed with H1N1 exposure. Therefore, a potential therapeutic strategy reduce the burden on influenza-related CVD may be to prescribe aspirin. There is an aversion to starting new aspirin therapy in influenza patients with the classic association of aspirin, influenza and Reye’s Syndrome (which does occur in adults) [43]. Retrospective evidence suggests patients who use aspirin have higher mortality compared to those who do not [44][45]; however, these associations may be confounded by concomitant risk factors associated with aspirin use. Recent studies have proposed mechanisms by which aspirin could have anti-viral and anti-inflammatory effects in influenza infections [46][47]. Therefore the effect of starting aspirin therapy for CVD risk reduction as well as influenza-related outcomes in the peri-influenza period is not known and warrants further study.

### Limitations

The primary limitation in the CATHGEN cohort is that we have no laboratory or clinical information of viral exposure or infection. As a consequence, we cannot confirm the molecular evidence of viral infection by viral GES with any laboratory/clinical data. Further, we do not know what type of respiratory virus patients may have been exposed to. This additional information could help explain the lack of correlation between the viral and platelet GES in the CATHGEN cohorts. We found evidence for primarily an H1N1 effect on the platelet GES in the infection cohorts. In contrast the viral GES is a non-specific respiratory viral infection classifier. Therefore, if there were a predominance of non-H1N1 viral exposure in CATHGEN patients then we would not expect to find a correlation between the viral and platelet GES. Second, the CATHGEN cohort study was performed as a cross sectional study; hence, we cannot know if the MI led to an increased viral GES or vice versa. Longitudinal studies could help provide evidence of causality. Third, it is possible that the changes in the viral GES could be, in part, due to 1) vaccination because 57% (n = 28/49) of genes in the viral GES do overlap with genes that change in response to influenza vaccination [48] or 2) concomitant medications used to prevent MI. Because the viral GES is an acute response signature, vaccination would have had to occur within days of catheterization and thus it is unlikely to confound the association we found between viral GES and MI. We assessed for an imbalance in concomitant medication use at the time of cardiac catheterization and found none between those with a positive vs. negative viral GES (Table 3). Last, influenza is associated with peri-/myocarditis, which clinically may mimic MI. Therefore a portion of our MI cases may have represented myocarditis and not thrombosis of a coronary artery. However, when we analyzed the STEMI subgroup, which is a clinical condition more clearly linked to thrombosis and less likely to be confused with pericarditis or myocarditis, we found a similar association with viral GES.

Among the viral exposure studies, the main limitation is that we did not measure traditional measures platelet function such as platelet aggregometry; however, we have previously shown that the platelet GES is a reproducible biomarker for platelet aggregation in response to aspirin [13].

In both the CATHGEN and infection cohorts, the samples sizes were relatively small, therefore independent validation in other cohorts is critical to confirm (or refute) our observations.

## Conclusions

A blood GES of viral infection was associated with MI. Separately, H1N1 exposure was associated with changes in a platelet GES that reflects higher levels of platelet function and an associated risk for MI. Together, these results highlight specific genes and pathways that explain the known relationship between viral infection, platelet activation, and MI especially in the case of H1N1 influenza infection.

.

## Supporting Information

S1 Fig Platelet GES probes_H1N1. (PDF)

S1 Table Numbers subjects with available RNA data at each time point after viral exposure in each viral cohort. (PDF)
