# Supplementary material for: Gene Expression Profiles Link Respiratory Viral Infection, Platelet Response to Aspirin, and Acute Myocardial Infarction
Source: PLoS One. 2015 Jul 20;10(7):e0132259. doi: 10.1371/journal.pone.0132259 (PMC4507878; doi:10.1371/journal.pone.0132259)
Supplement: S1 Fig — (PDF) [file pone.0132259.s001.pdf]

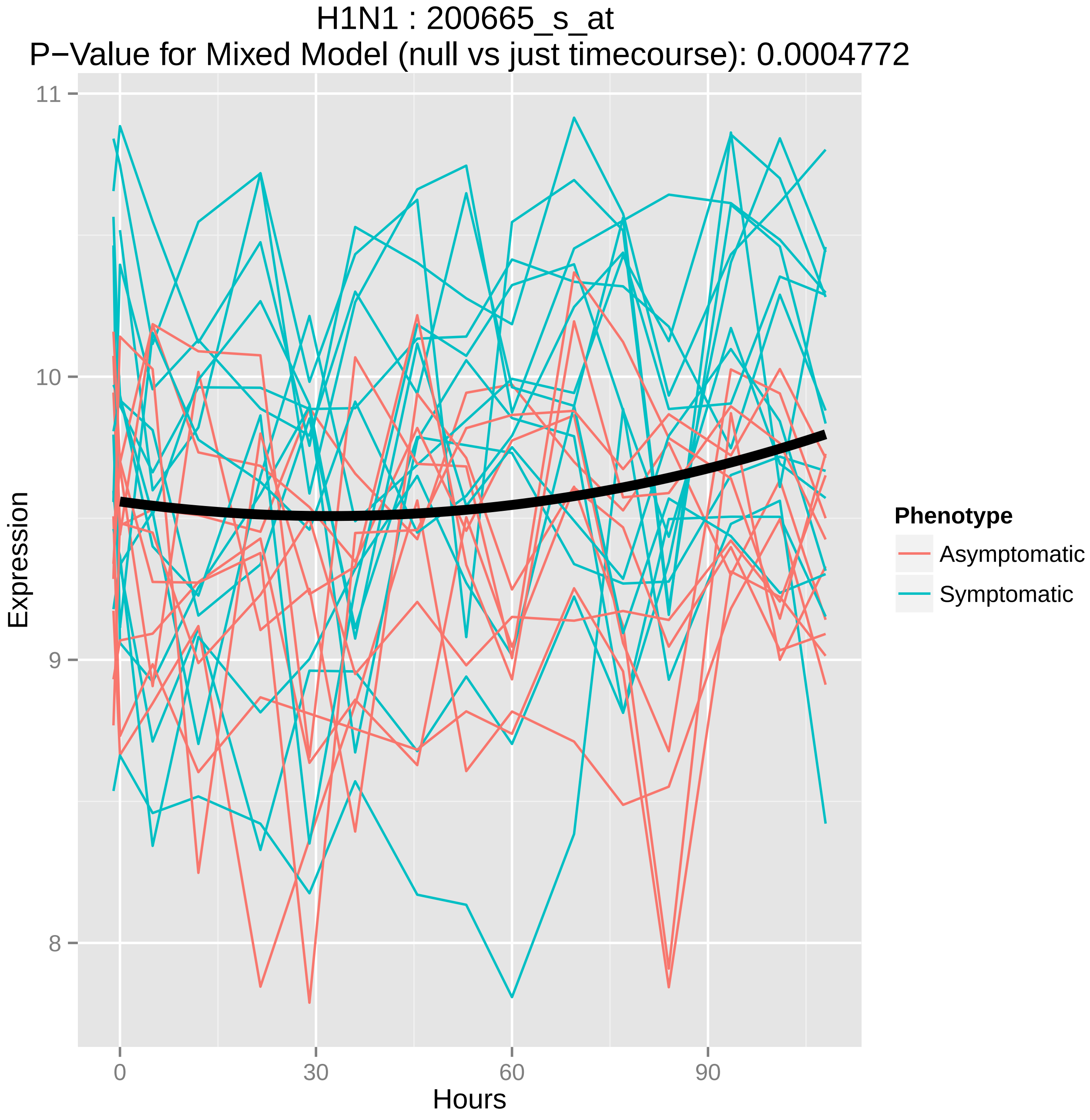

H1N1 : 200665_s_at
P−Value for Mixed Model (null vs just timecourse): 0.0004772
11
10
9
8
Expression
0
30
60
90
Hours
Phenotype
Asymptomatic
Symptomatic

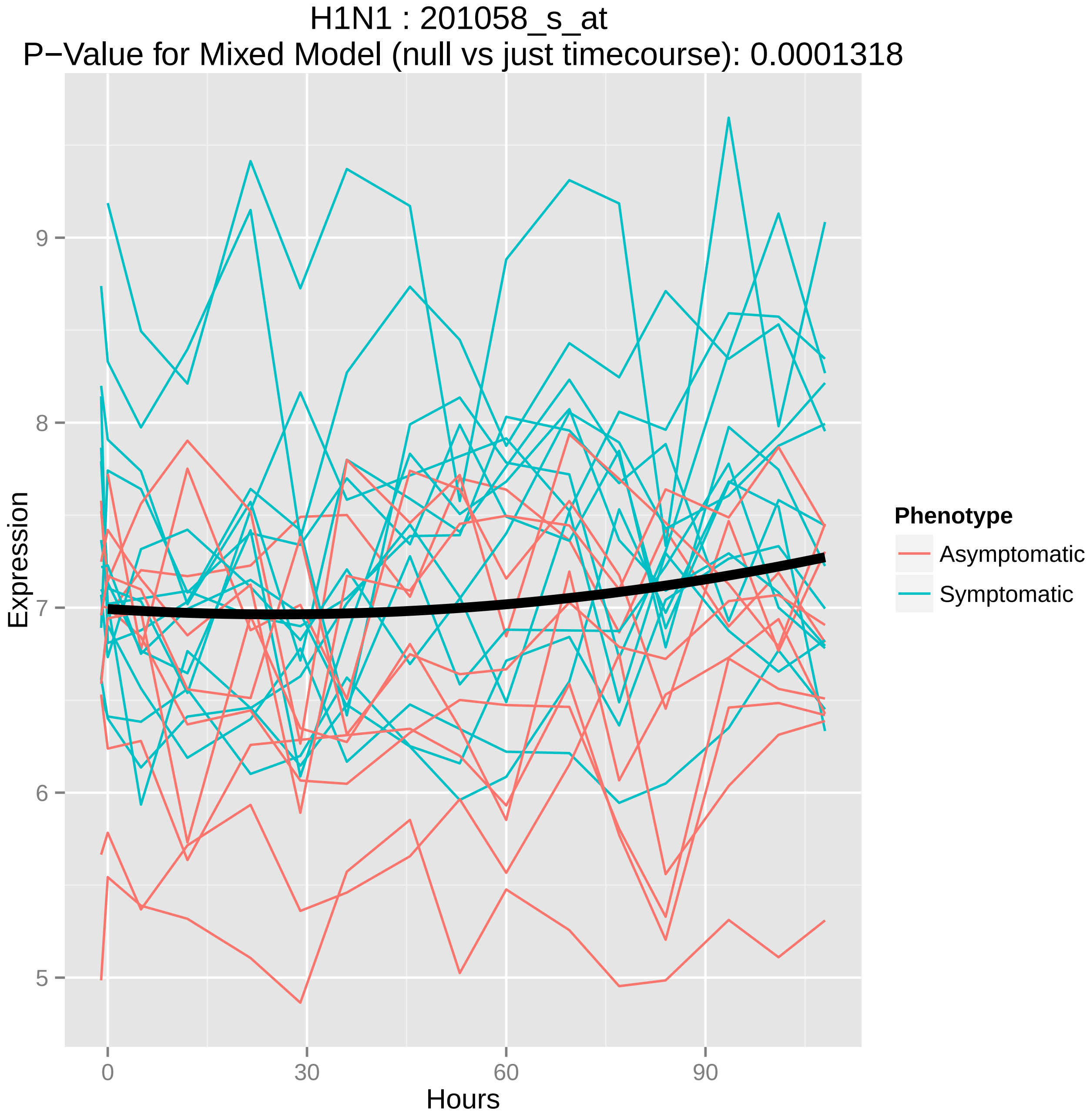
H1N1 : 201058_s_at
P−Value for Mixed Model (null vs just timecourse): 0.0001318
Expression
9
8
7
6
5
0
30
60
90
Hours
Phenotype
Asymptomatic
Symptomatic

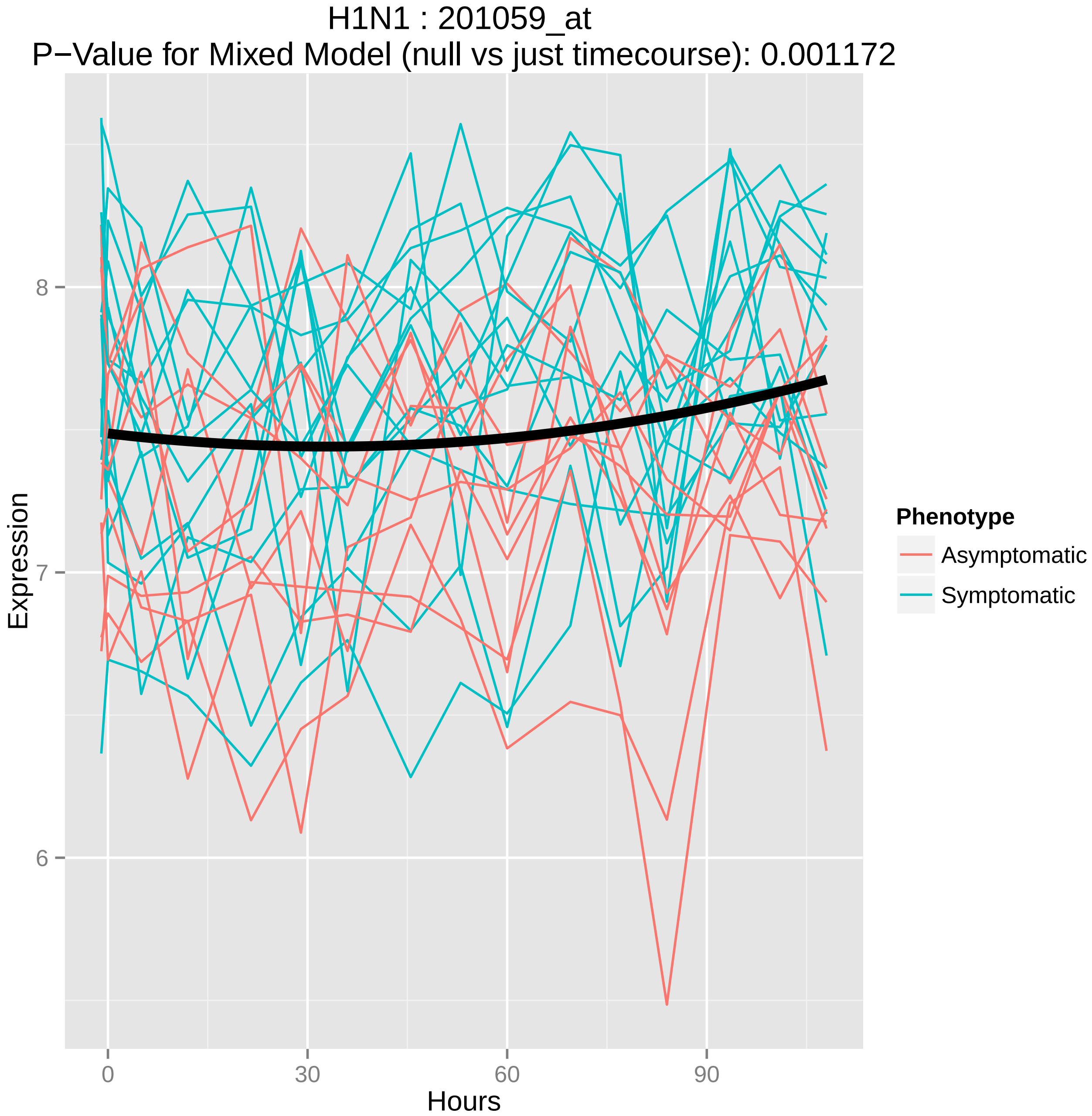

H1N1 : 201059_at
P−Value for Mixed Model (null vs just timecourse): 0.001172
Expression
8
7
6
0
30
60
90
Hours
Phenotype
Asymptomatic
Symptomatic

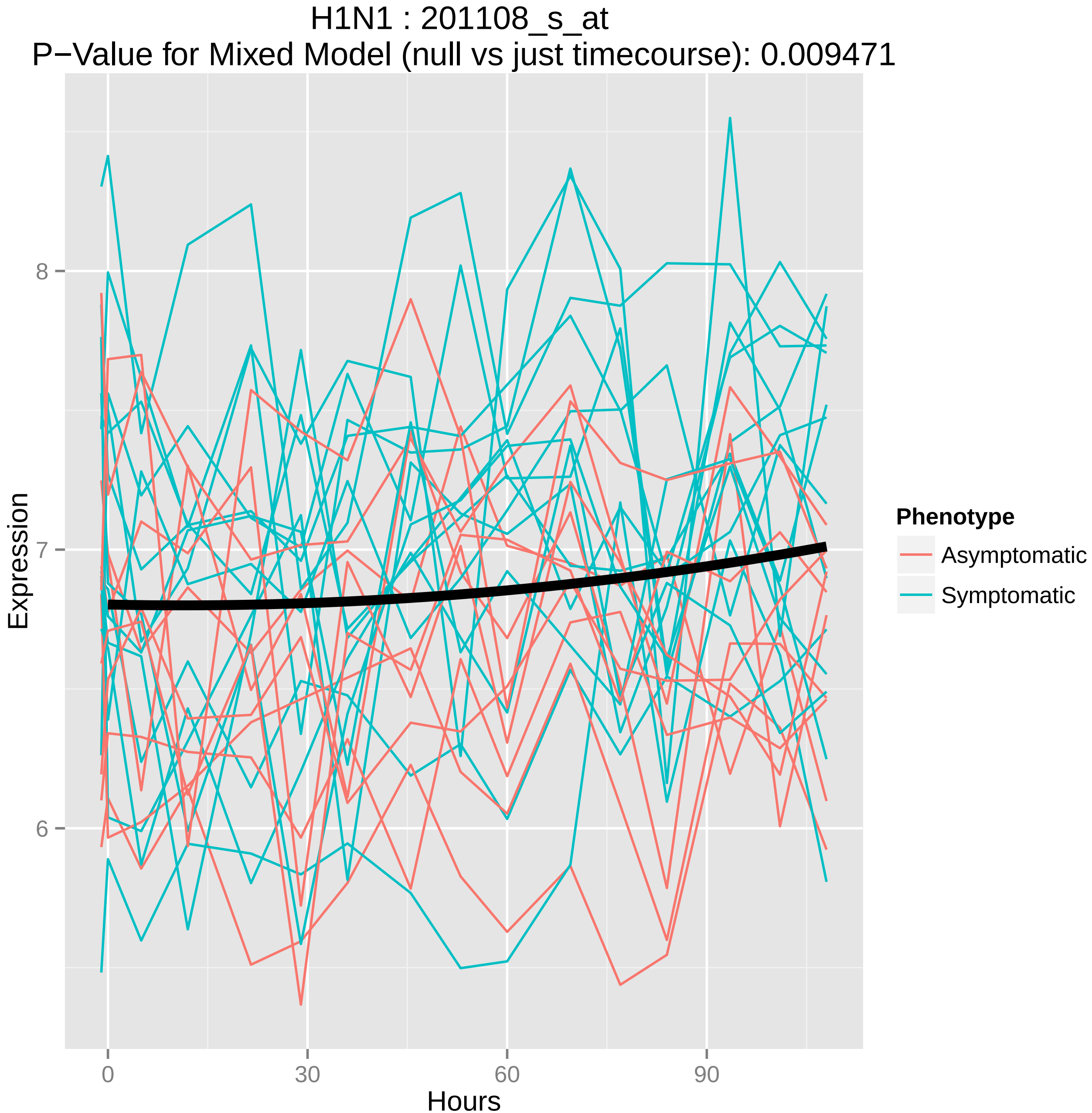
H1N1 : 201108_s_at
P−Value for Mixed Model (null vs just timecourse): 0.009471
Expression
8
7
6
Hours
0
30
60
90
Phenotype
Asymptomatic
Symptomatic

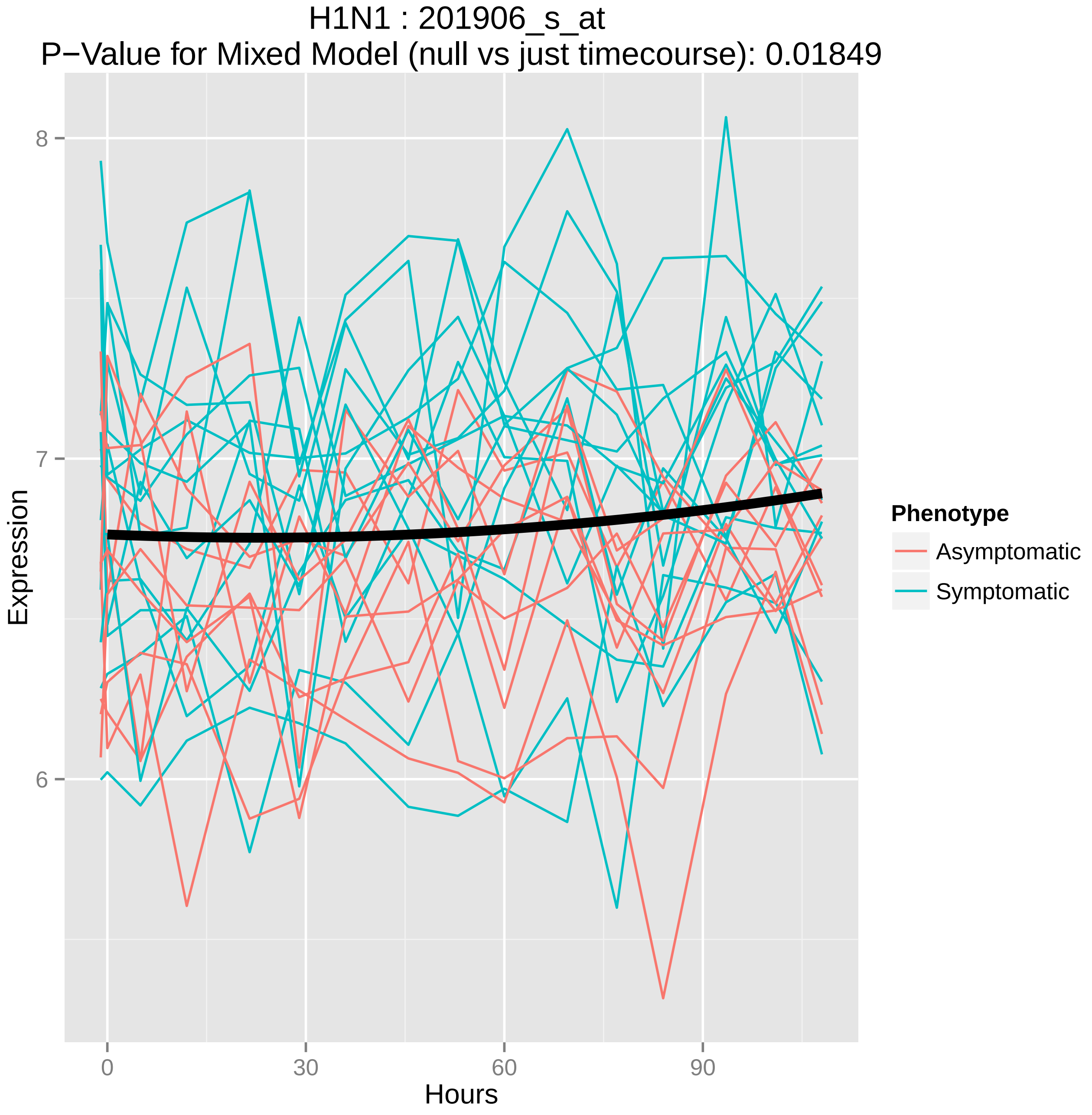
H1N1 : 201906_s_at
P−Value for Mixed Model (null vs just timecourse): 0.01849
Expression
8
7
6
0
30
60
90
Hours
Phenotype
Asymptomatic
Symptomatic

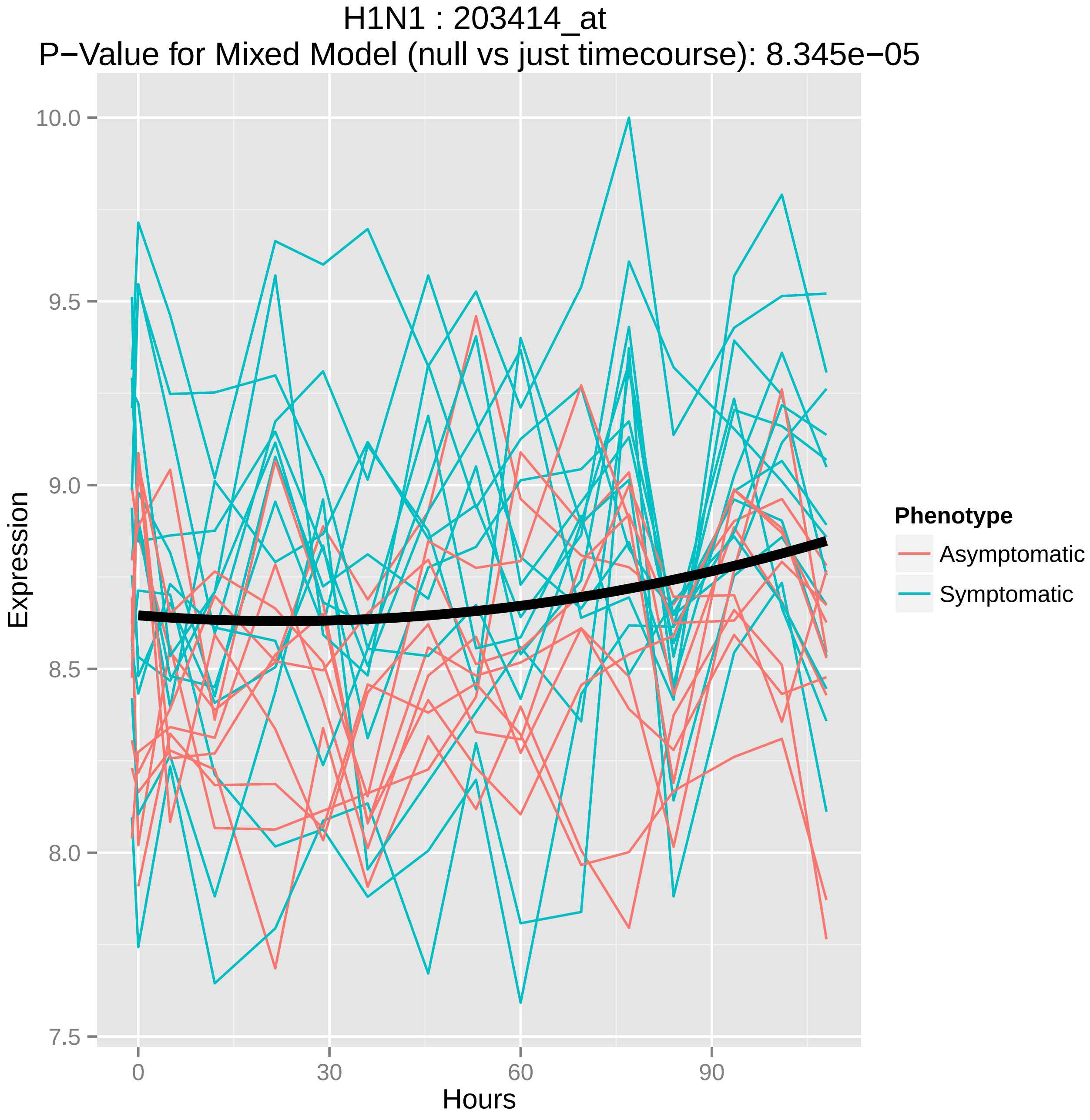
H1N1 : 203414_at
P−Value for Mixed Model (null vs just timecourse): 8.345e−05
Expression
10.0
9.5
9.0
8.5
8.0
7.5
0
30
60
90
Hours
Phenotype
Asymptomatic
Symptomatic

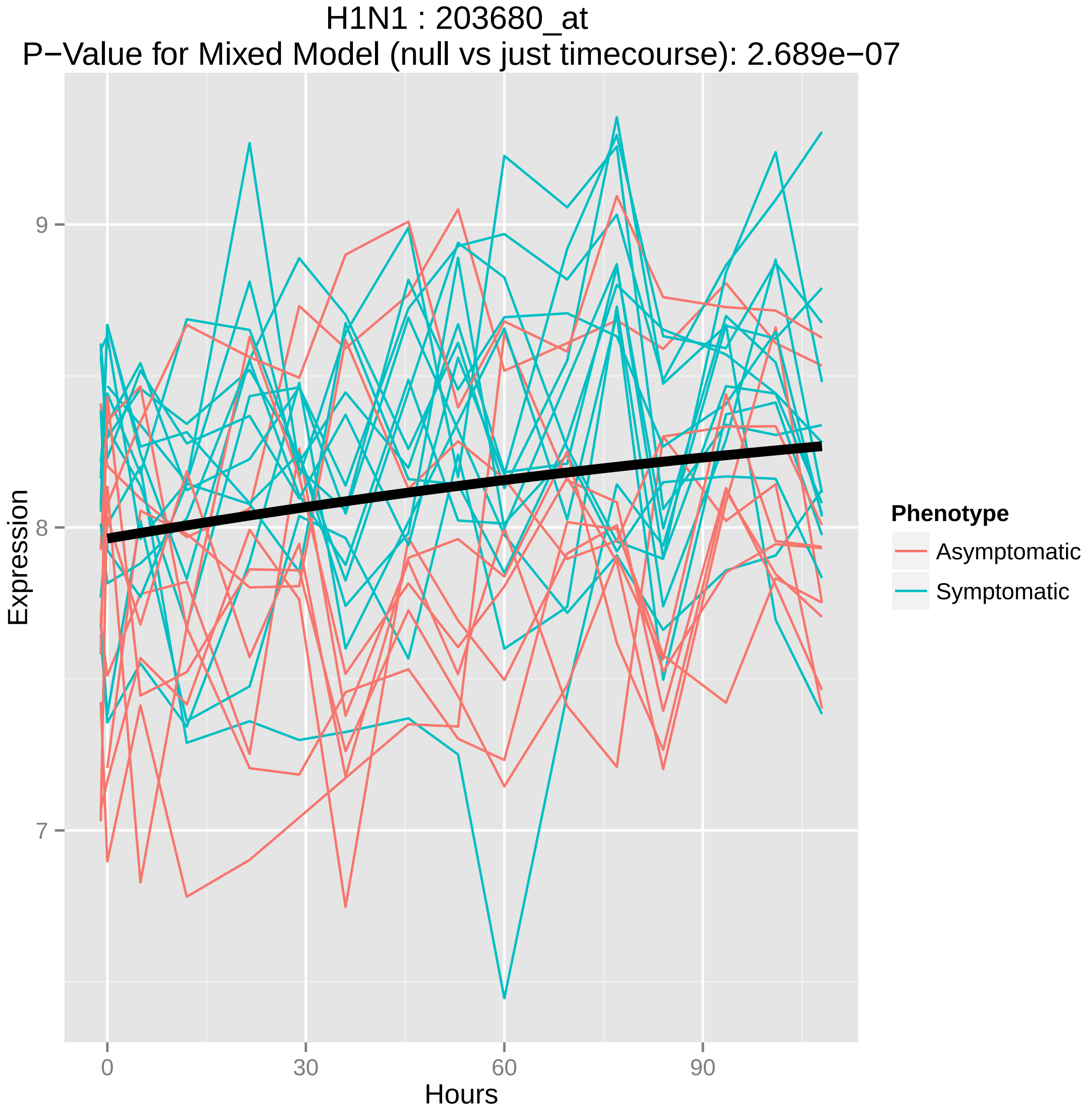
H1N1 : 203680_at
P−Value for Mixed Model (null vs just timecourse): 2.689e−07
Expression
9
8
7
0
30
60
90
Hours
Phenotype
Asymptomatic
Symptomatic

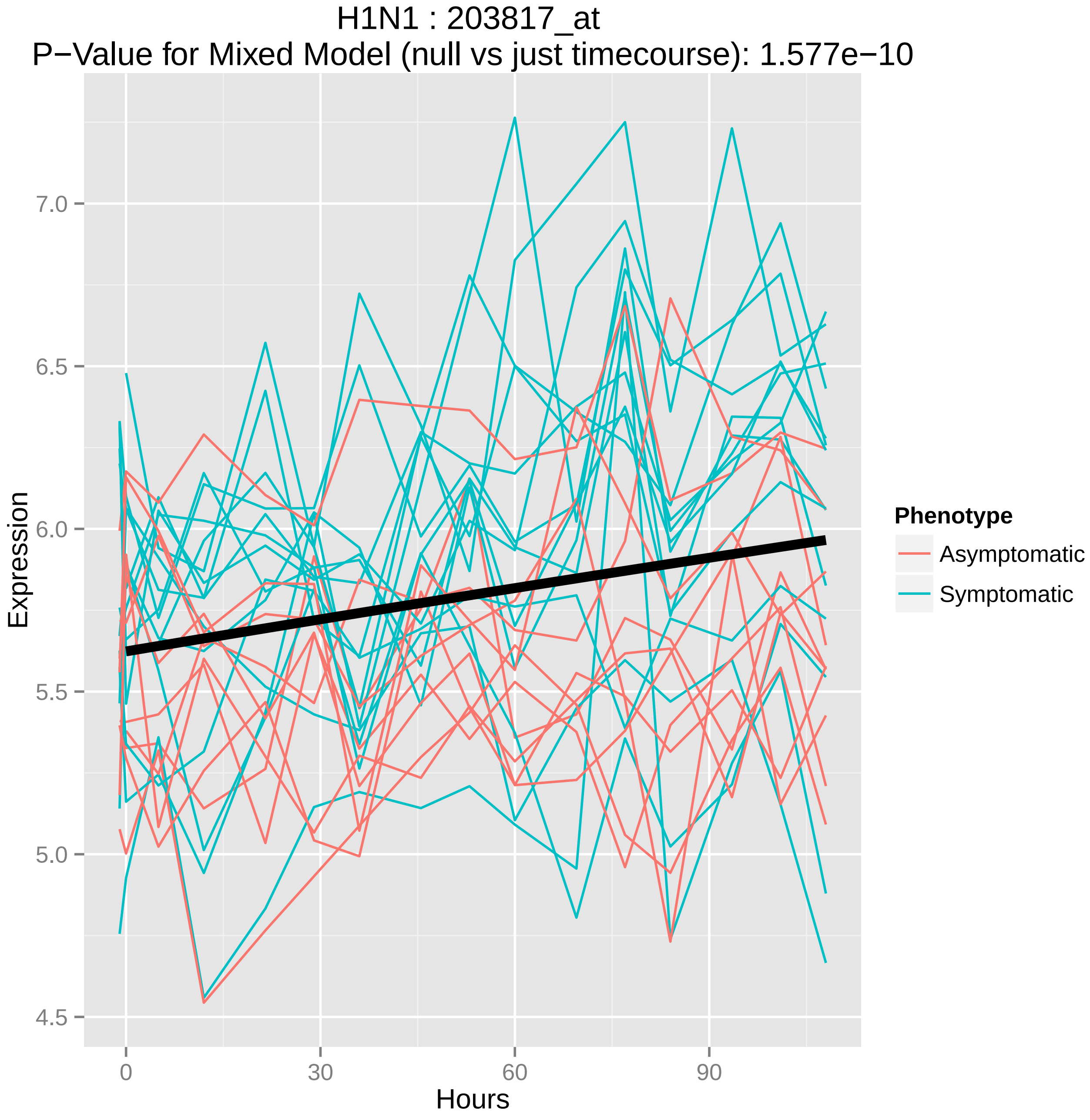
H1N1 : 203817_at
P−Value for Mixed Model (null vs just timecourse): 1.577e−10
Expression
7.0
6.5
6.0
5.5
5.0
4.5
0
30
60
90
Hours
Phenotype
Asymptomatic
Symptomatic

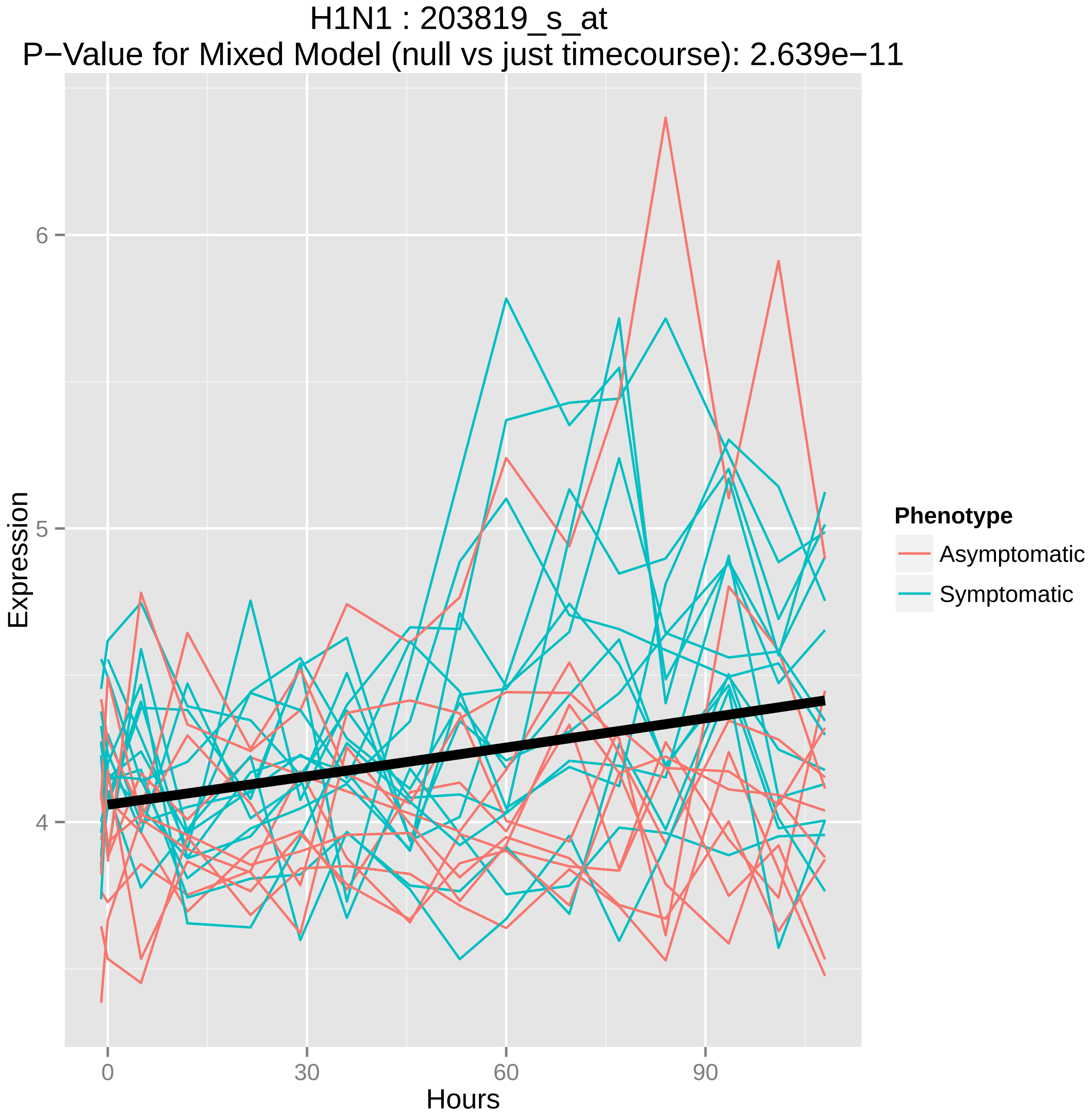

H1N1 : 203819_s_at
P−Value for Mixed Model (null vs just timecourse): 2.639e−11
Expression
6
5
4
0
30
60
90
Hours
Phenotype
Asymptomatic
Symptomatic

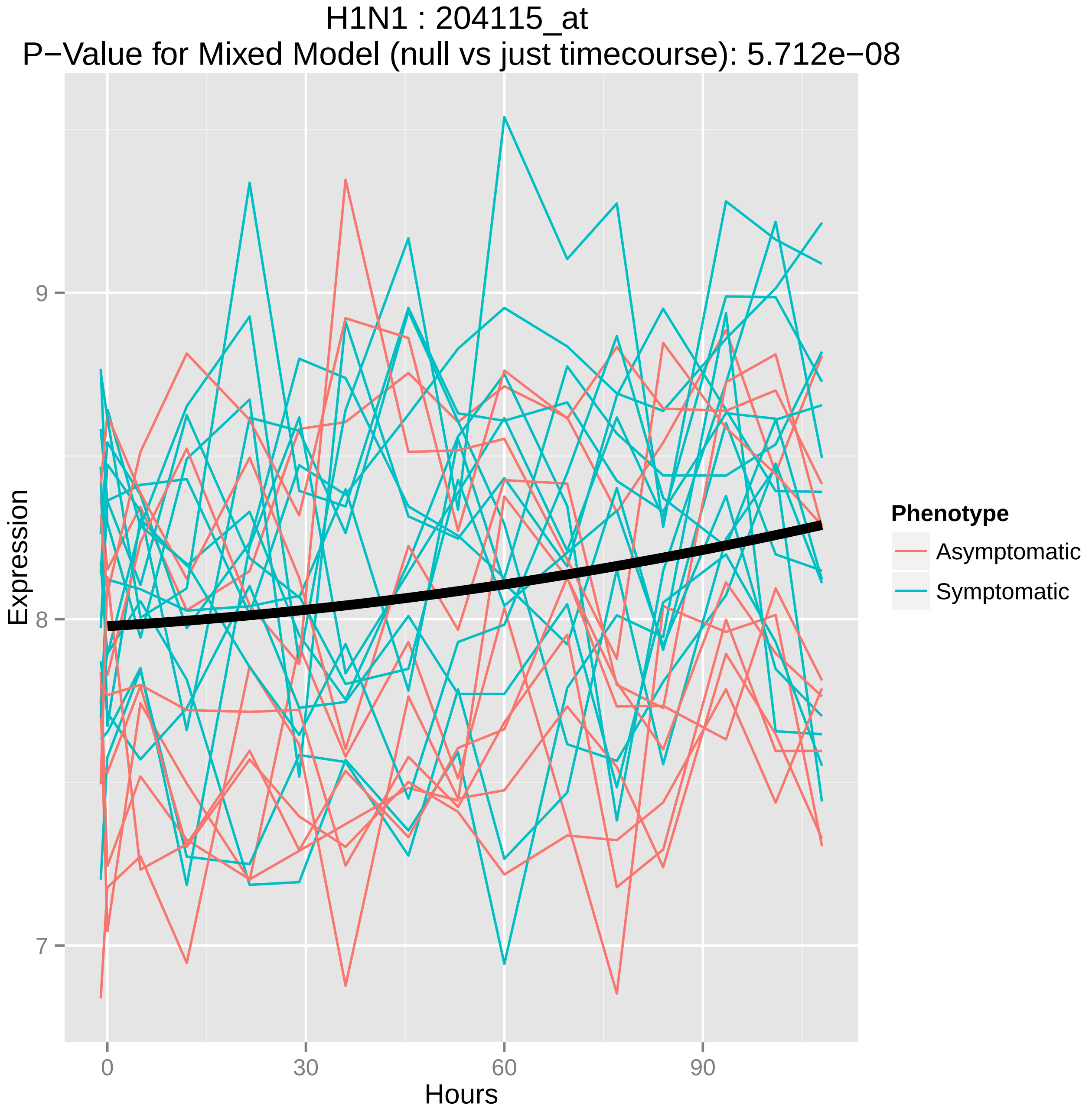

H1N1 : 204115_at
P−Value for Mixed Model (null vs just timecourse): 5.712e−08
Expression
Hours
9
8
7
0
30
60
90
Phenotype
Asymptomatic
Symptomatic

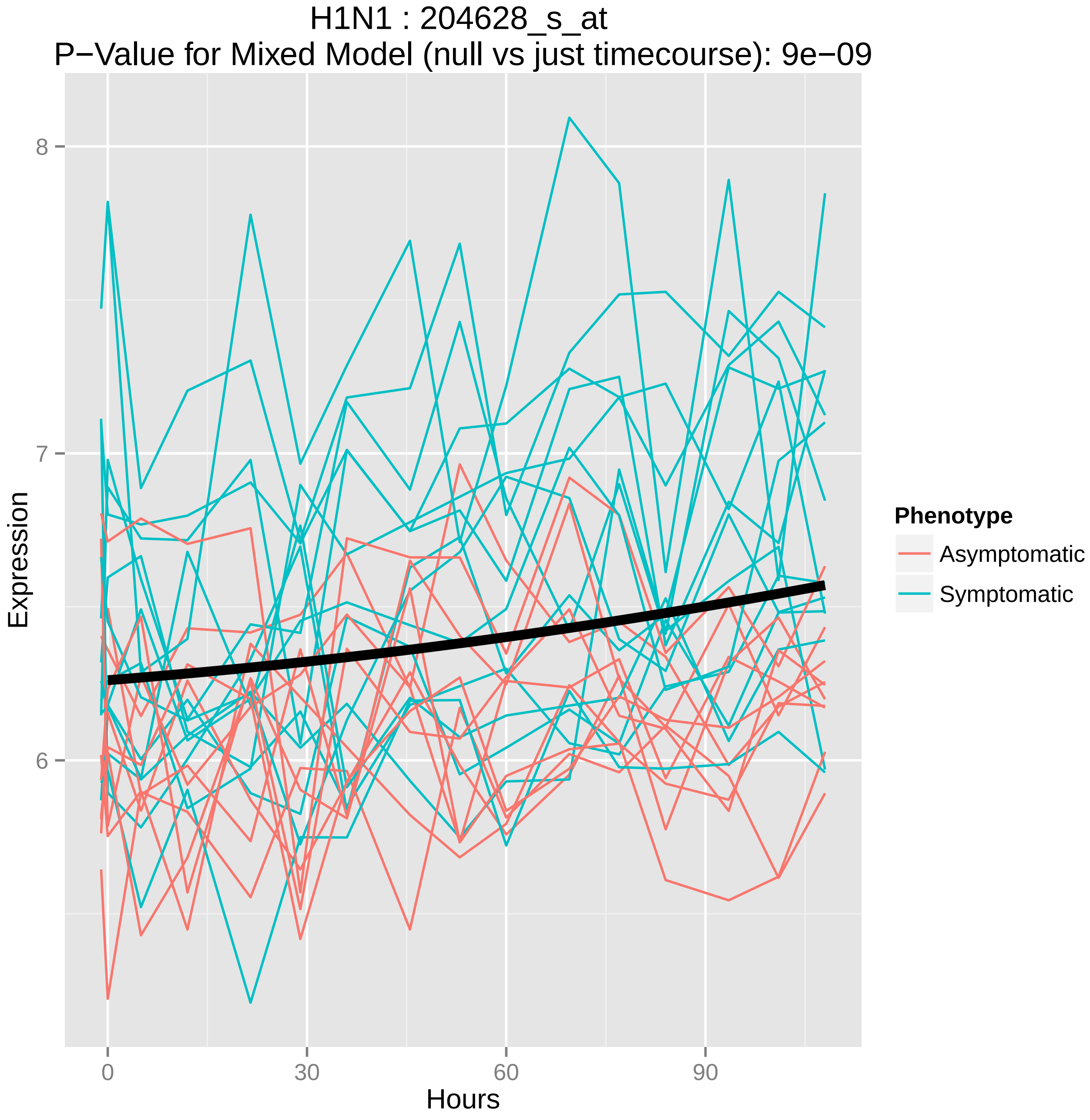

H1N1 : 204628_s_at
P−Value for Mixed Model (null vs just timecourse): 9e−09
Expression
8
7
6
0
30
60
90
Hours
Phenotype
Asymptomatic
Symptomatic

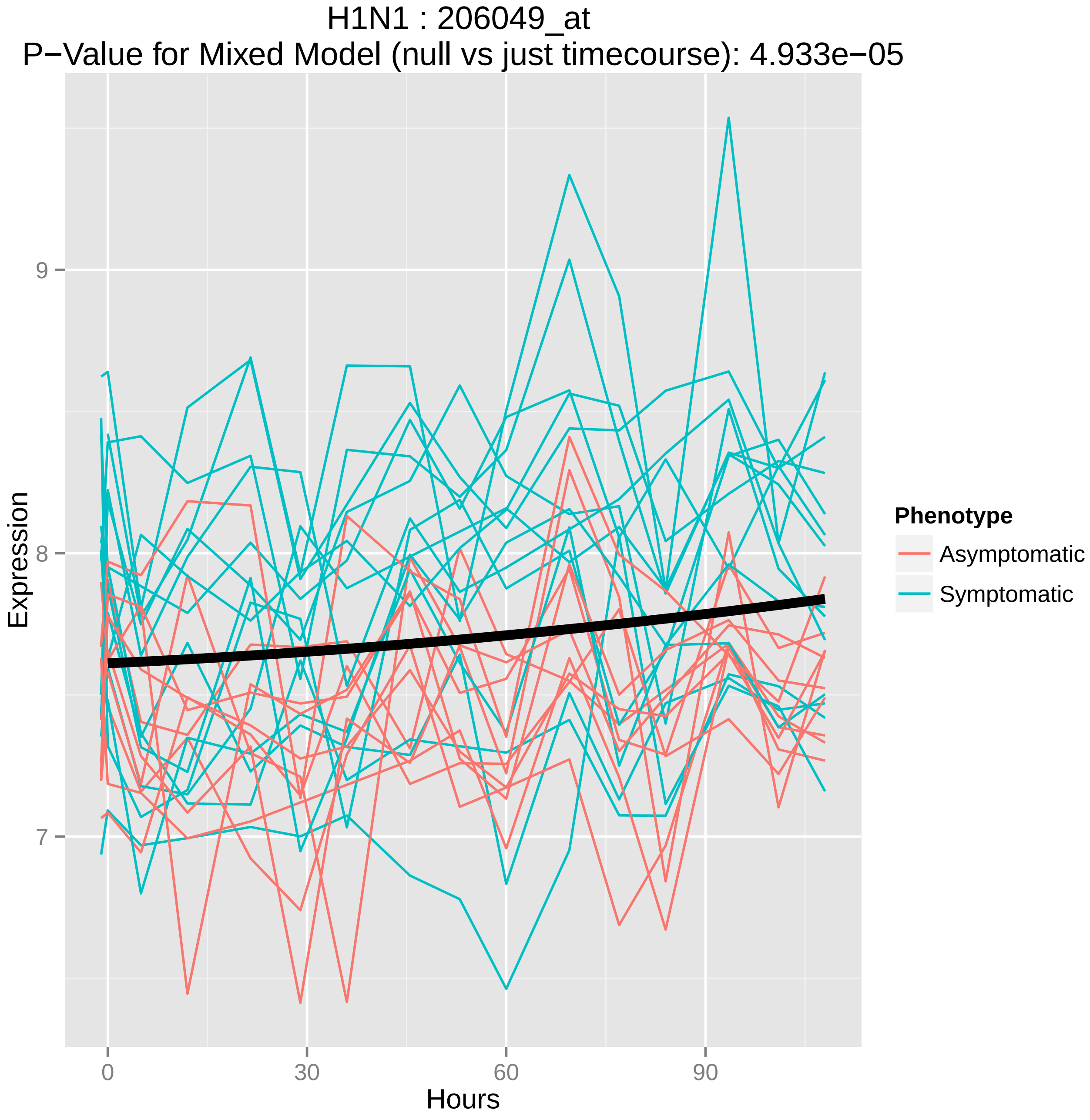

H1N1 : 206049_at
P−Value for Mixed Model (null vs just timecourse): 4.933e−05
Expression
Hours
9
8
7
0
30
60
90
Phenotype
Asymptomatic
Symptomatic

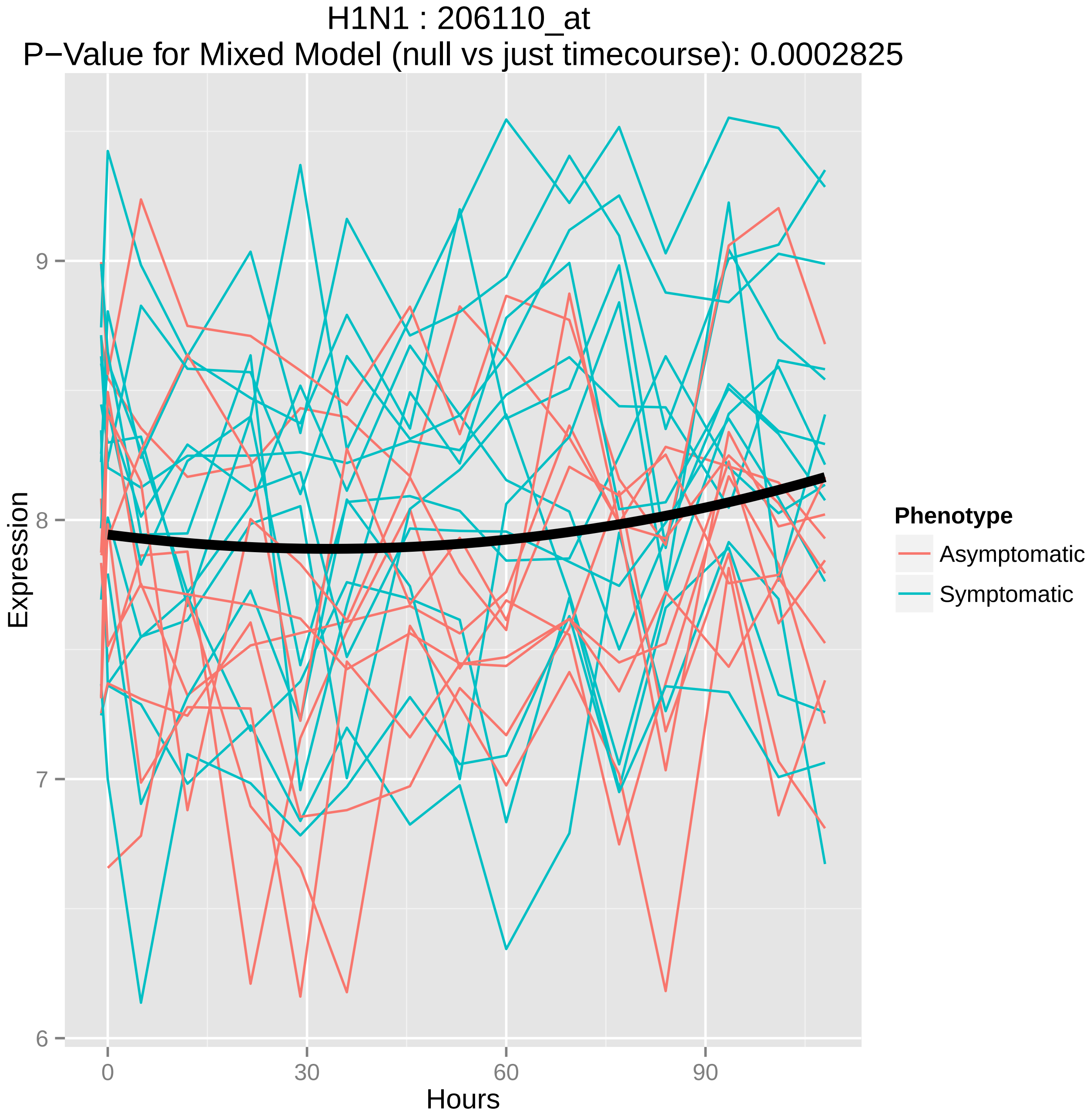

H1N1 : 206110_at
P−Value for Mixed Model (null vs just timecourse): 0.0002825
Expression
9
8
7
6
0
30
60
90
Hours
Phenotype
Asymptomatic
Symptomatic

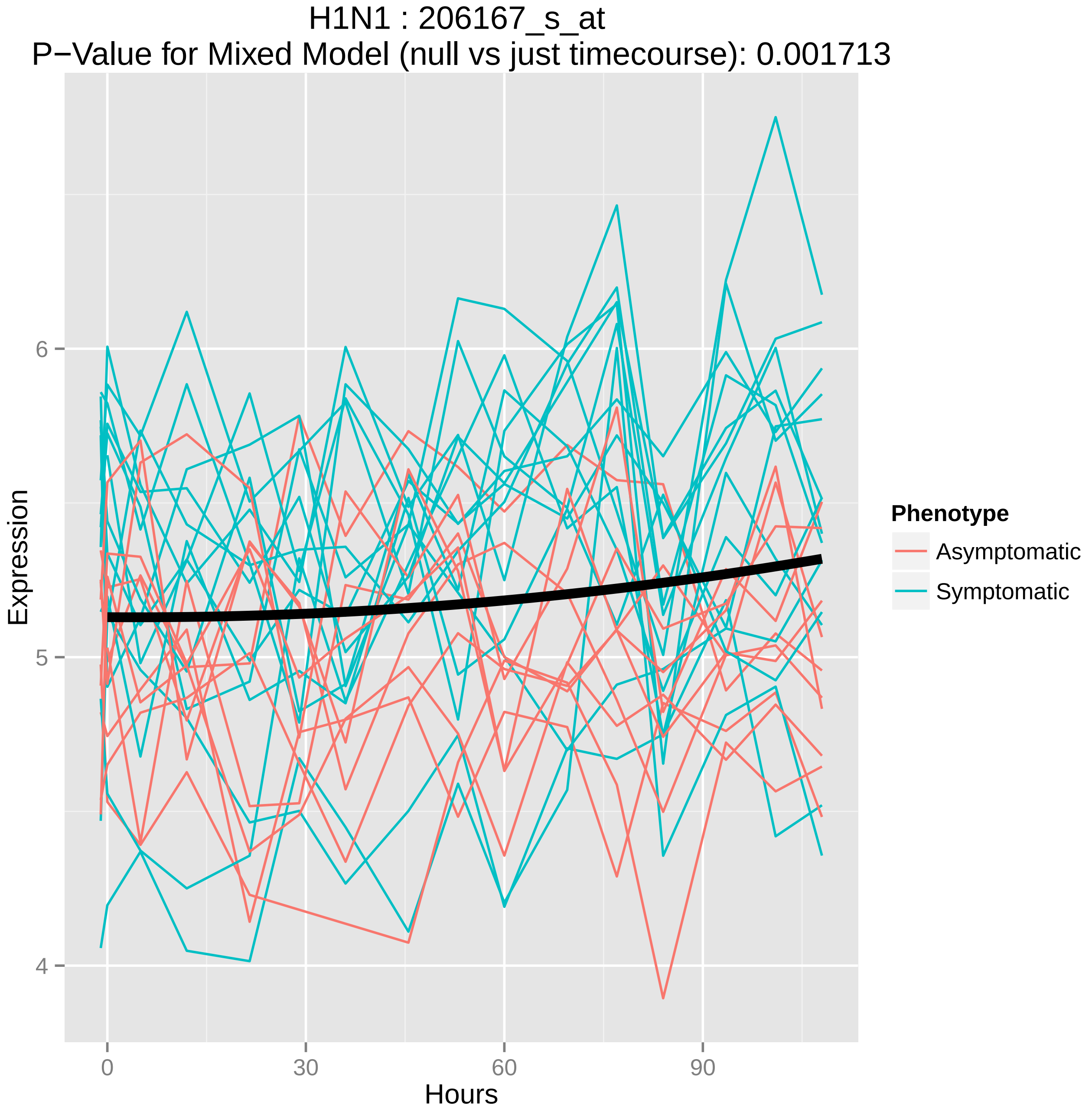

H1N1 : 206167_s_at
P−Value for Mixed Model (null vs just timecourse): 0.001713
Expression
6
5
4
0
30
60
90
Hours
Phenotype
Asymptomatic
Symptomatic

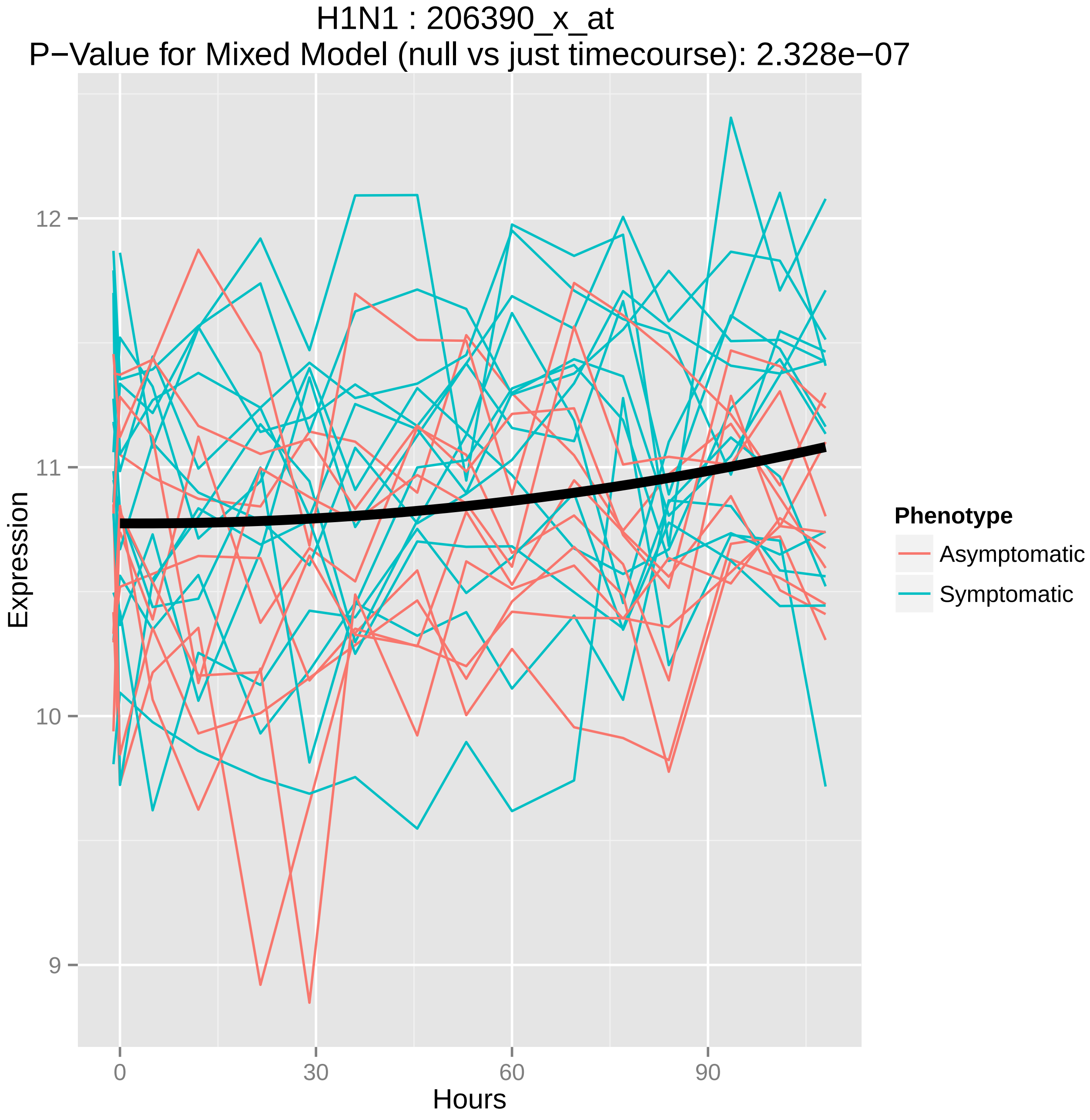

H1N1 : 206390_x_at
P−Value for Mixed Model (null vs just timecourse): 2.328e−07
Expression
12
11
10
9
0
30
60
90
Hours
Phenotype
Asymptomatic
Symptomatic

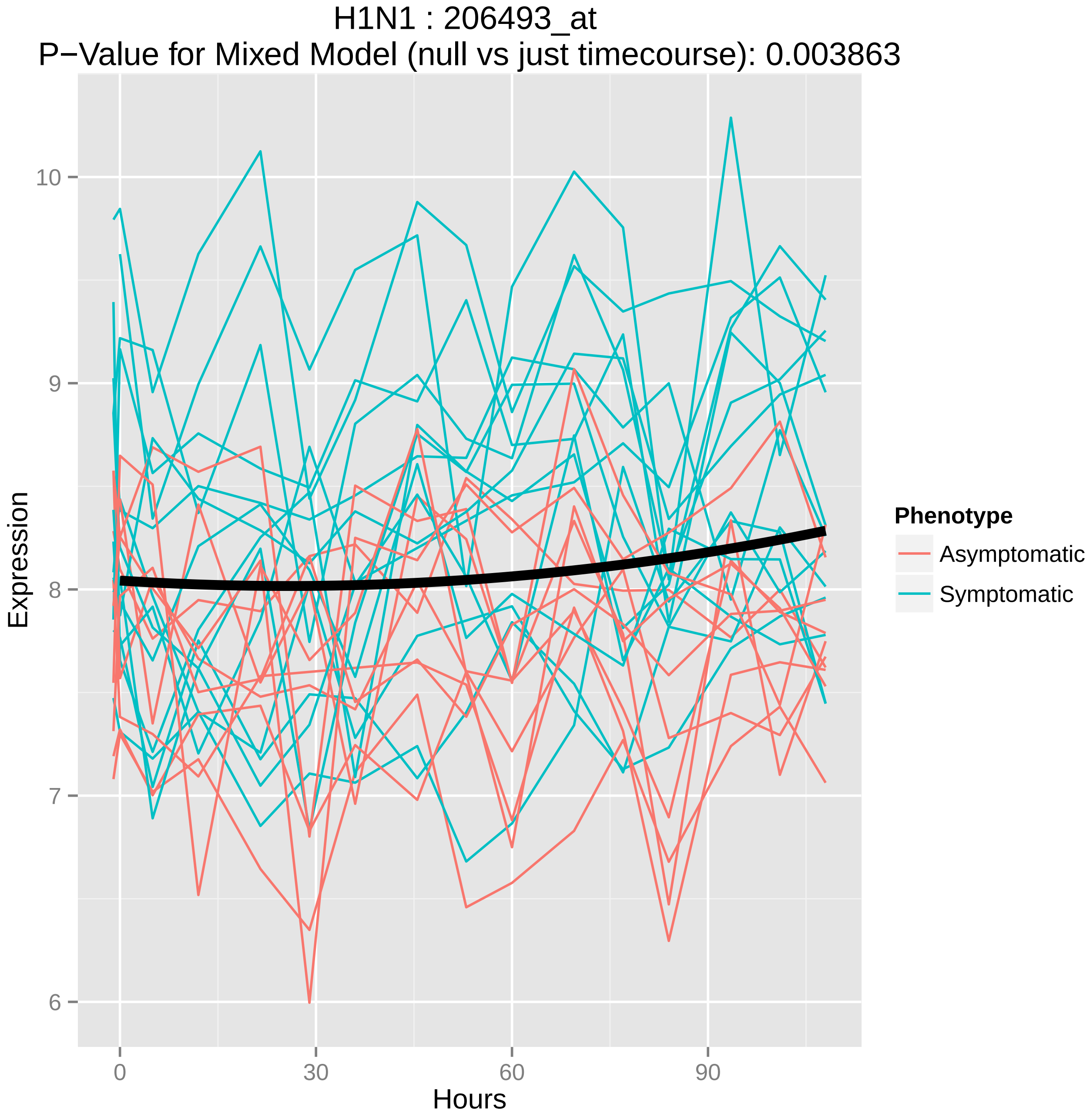
H1N1 : 206493_at
P−Value for Mixed Model (null vs just timecourse): 0.003863
Expression
10
9
8
7
6
0
30
60
90
Hours
Phenotype
Asymptomatic
Symptomatic

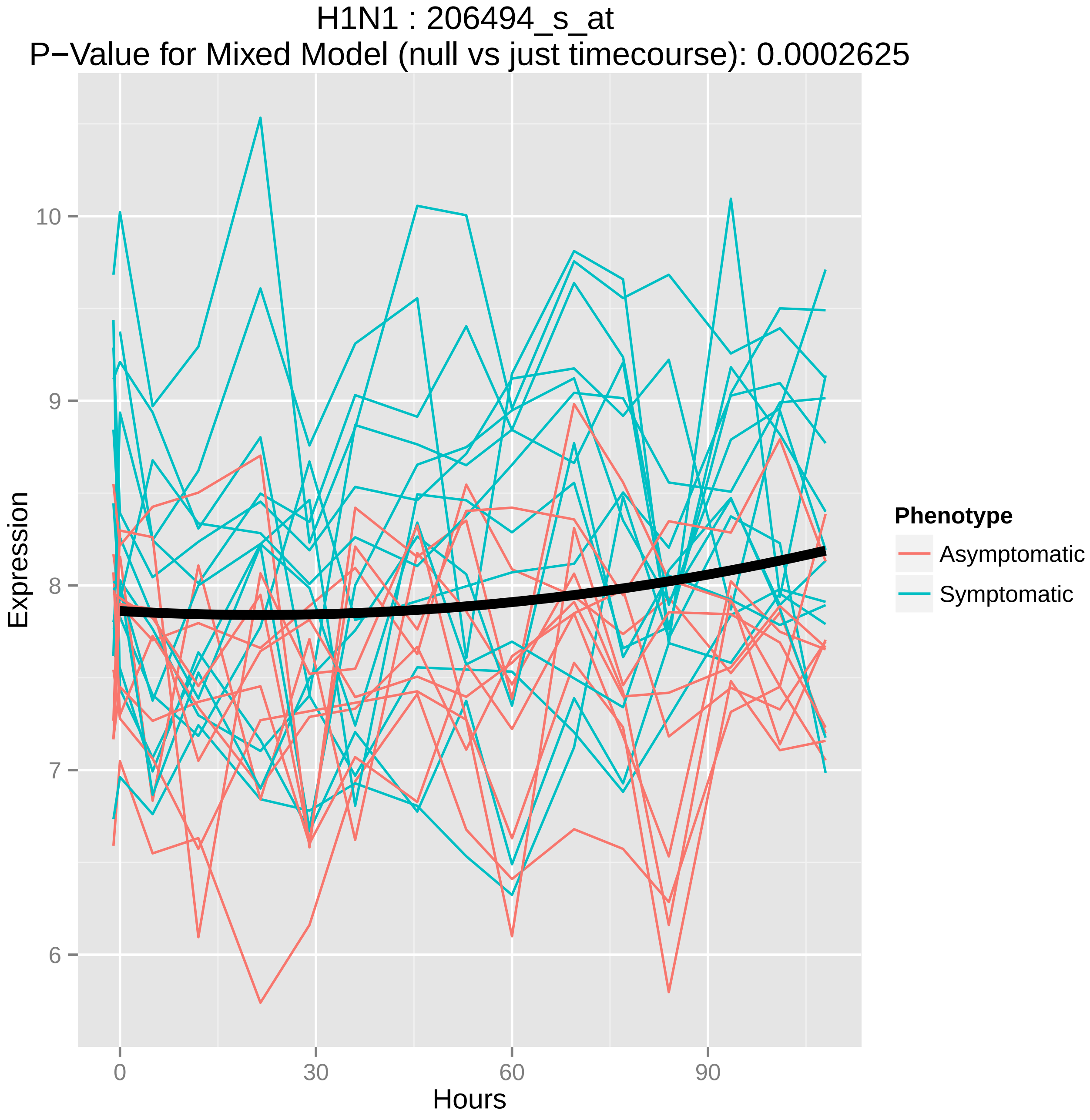
H1N1 : 206494_s_at
P−Value for Mixed Model (null vs just timecourse): 0.0002625
Expression
10
9
8
7
6
0
30
60
90
Hours
Phenotype
Asymptomatic
Symptomatic

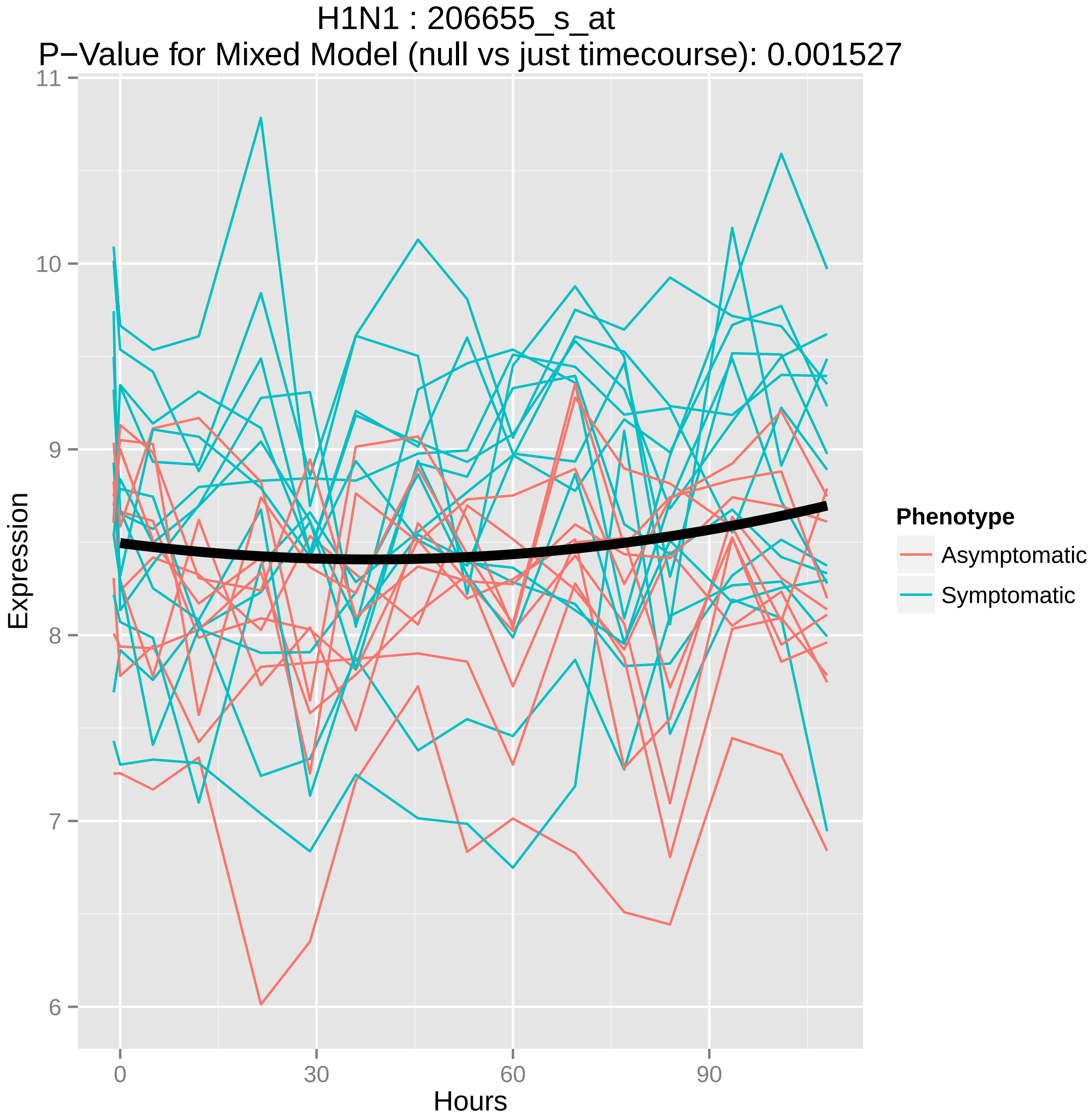

H1N1 : 206655_s_at
P−Value for Mixed Model (null vs just timecourse): 0.001527
Expression
Hours
11
10
9
8
7
6
0
30
60
90
Phenotype
Asymptomatic
Symptomatic

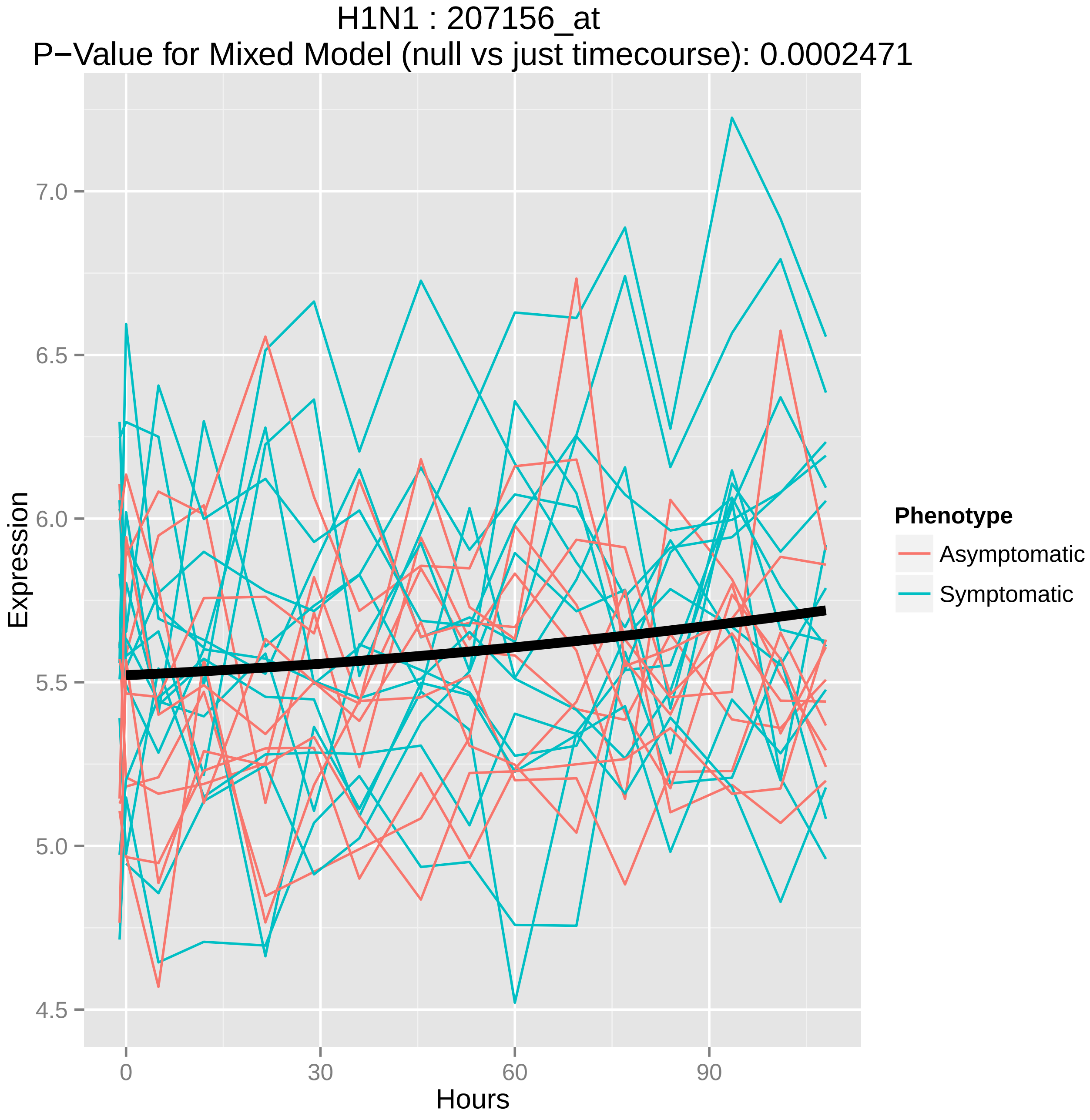
H1N1 : 207156_at
P−Value for Mixed Model (null vs just timecourse): 0.0002471
Expression
7.0
6.5
6.0
5.5
5.0
4.5
0
30
60
90
Hours
Phenotype
Asymptomatic
Symptomatic

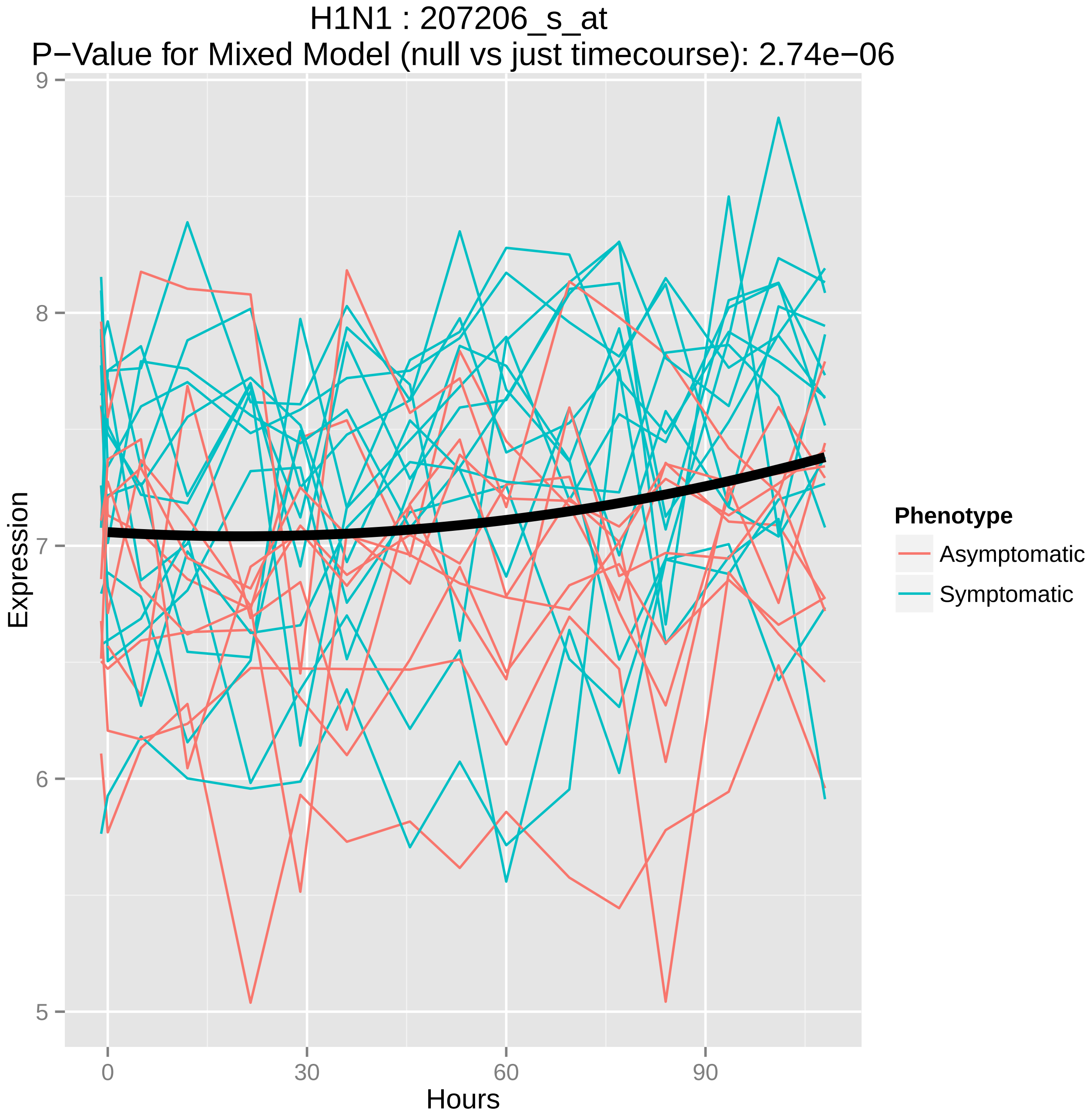

H1N1 : 207206_s_at
P−Value for Mixed Model (null vs just timecourse): 2.74e−06
Expression
Hours
9
8
7
6
5
0
30
60
90
Phenotype
Asymptomatic
Symptomatic

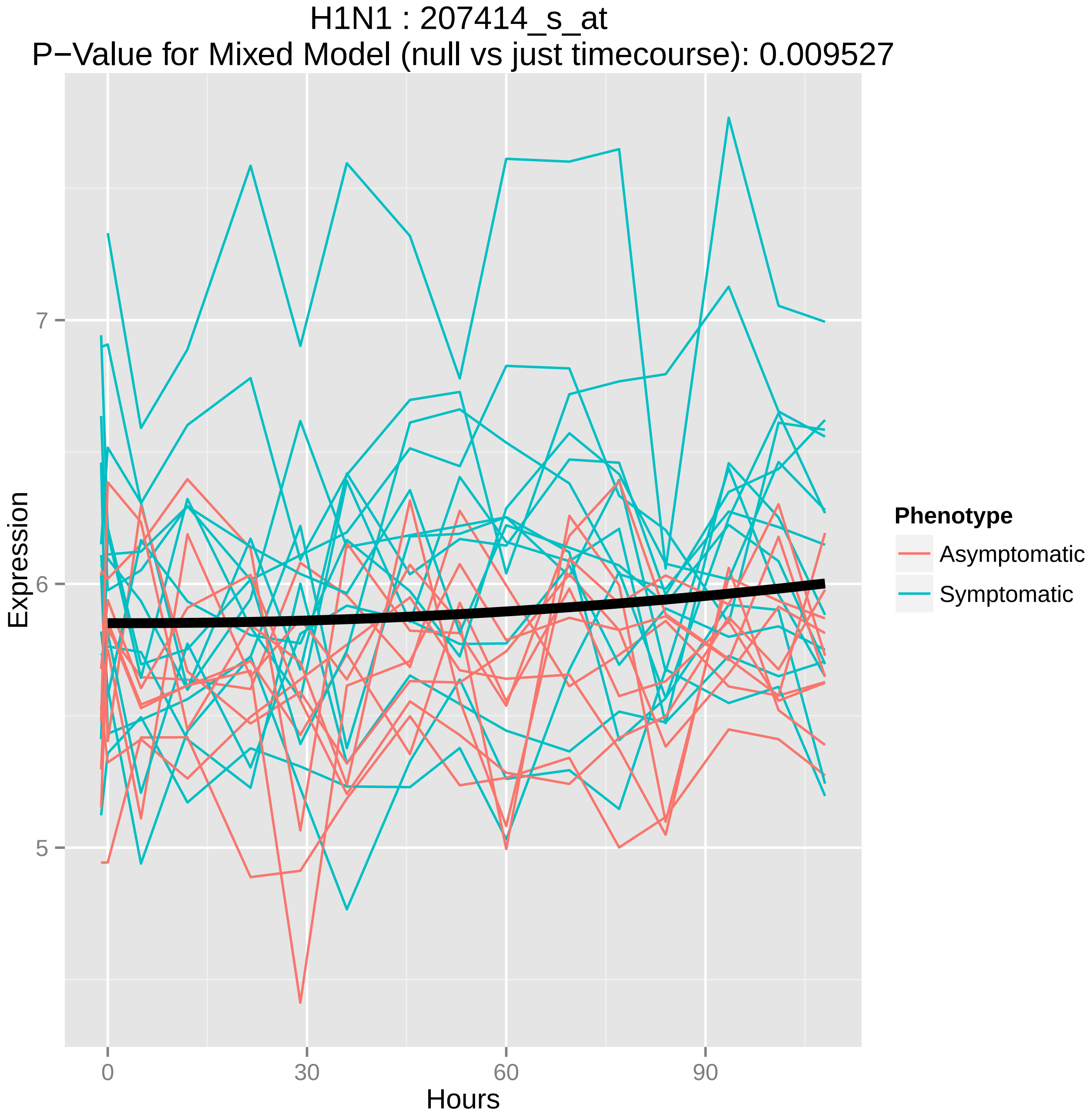
H1N1 : 207414_s_at
P−Value for Mixed Model (null vs just timecourse): 0.009527
Expression
7
6
5
0
30
60
90
Hours
Phenotype
Asymptomatic
Symptomatic

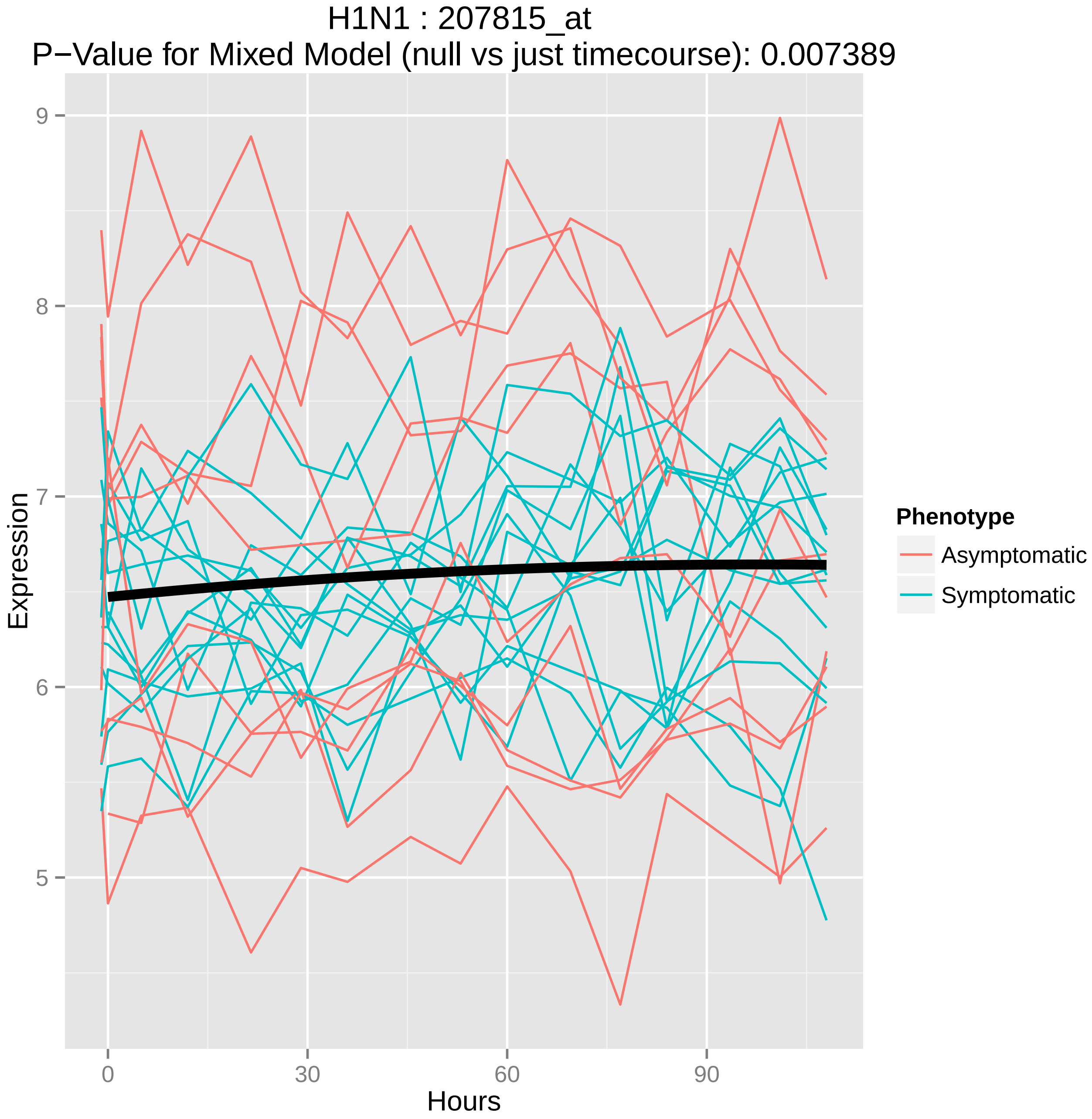

H1N1 : 207815_at
P−Value for Mixed Model (null vs just timecourse): 0.007389
Expression
Hours
9
8
7
6
5
0
30
60
90
Phenotype
Asymptomatic
Symptomatic

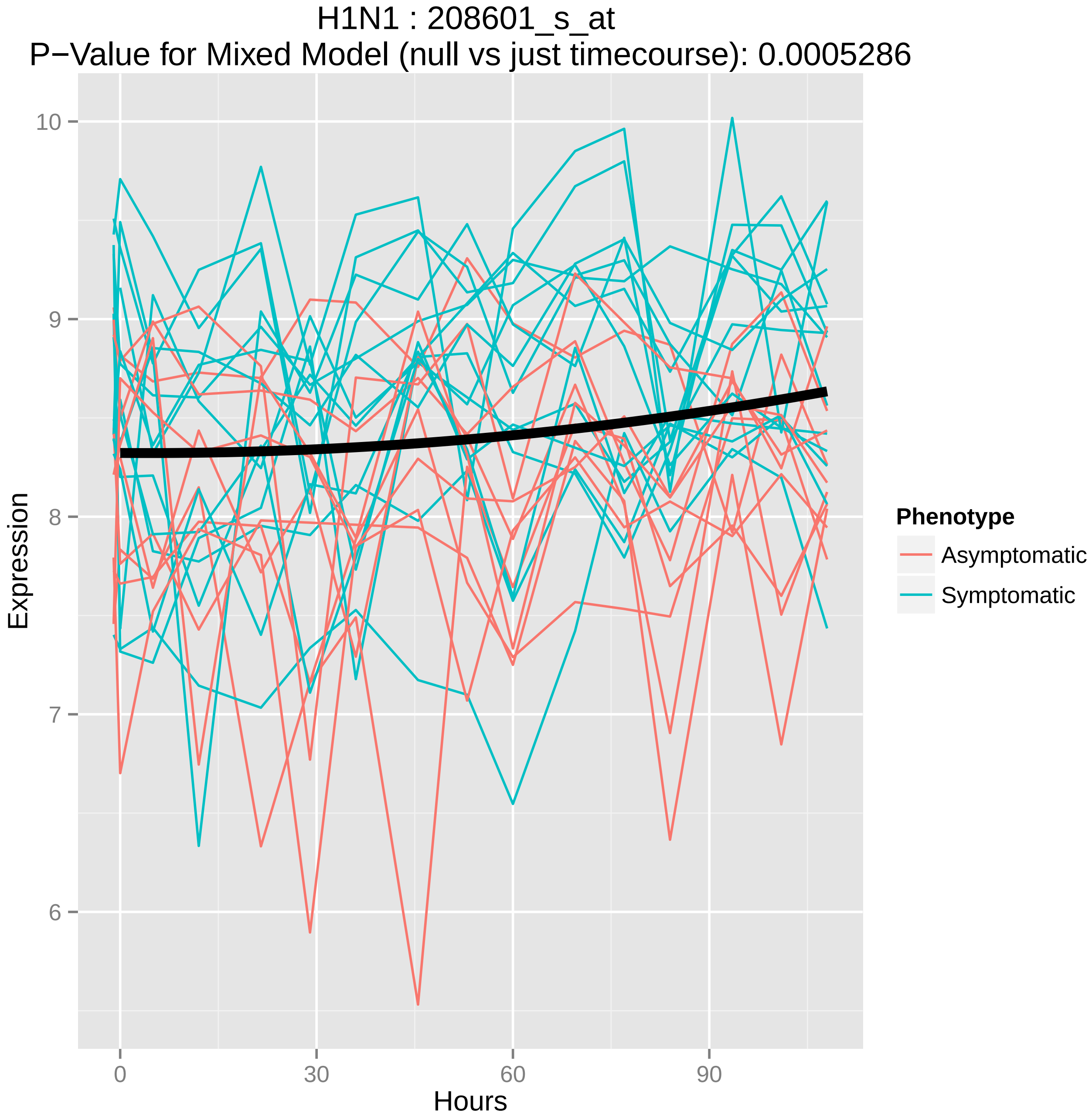
H1N1 : 208601_s_at
P−Value for Mixed Model (null vs just timecourse): 0.0005286
Expression
10
9
8
7
6
0
30
60
90
Hours
Phenotype
Asymptomatic
Symptomatic

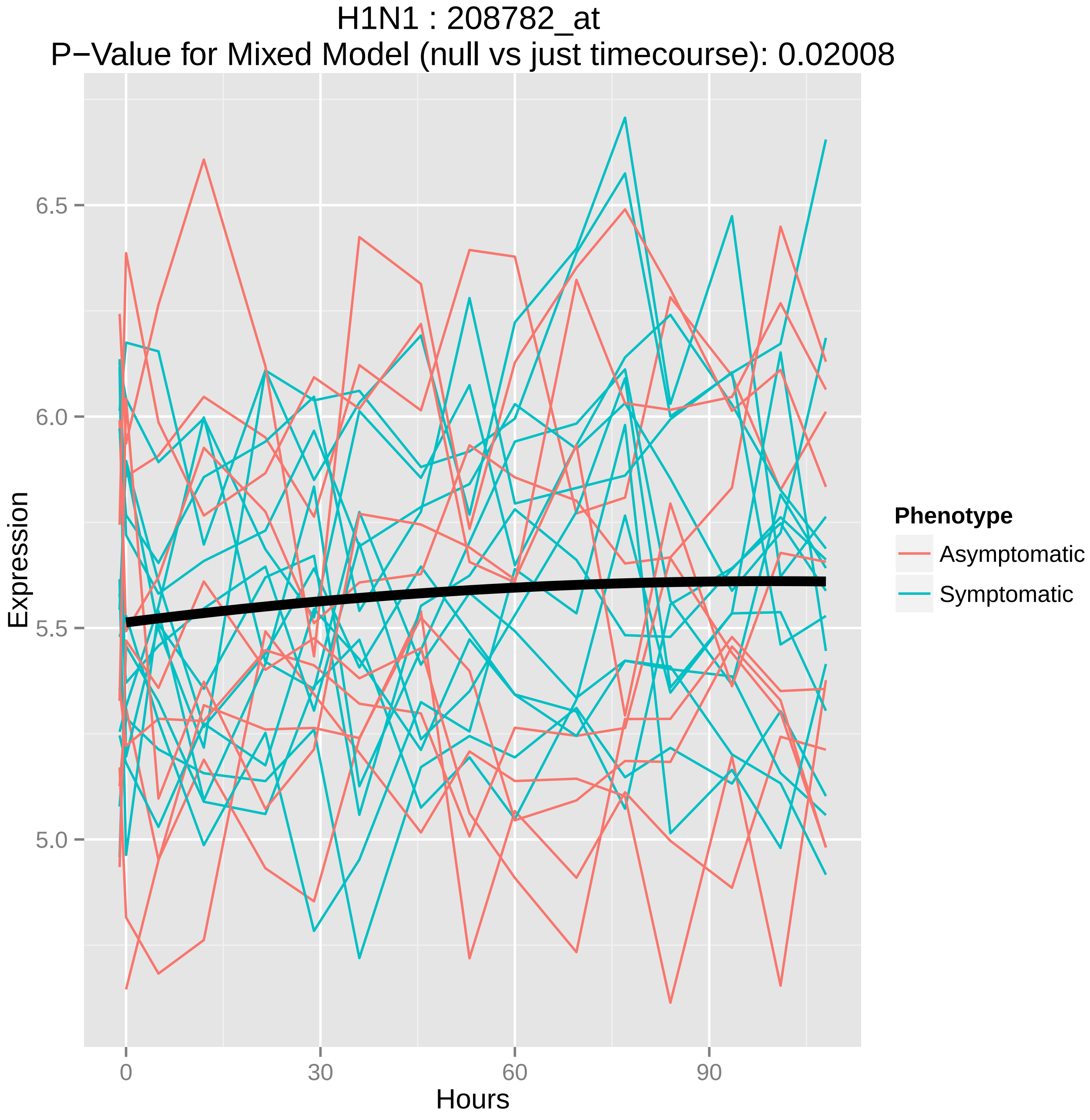

H1N1 : 208782_at
P−Value for Mixed Model (null vs just timecourse): 0.02008
Expression
6.5
6.0
5.5
5.0
0
30
60
90
Hours
Phenotype
Asymptomatic
Symptomatic

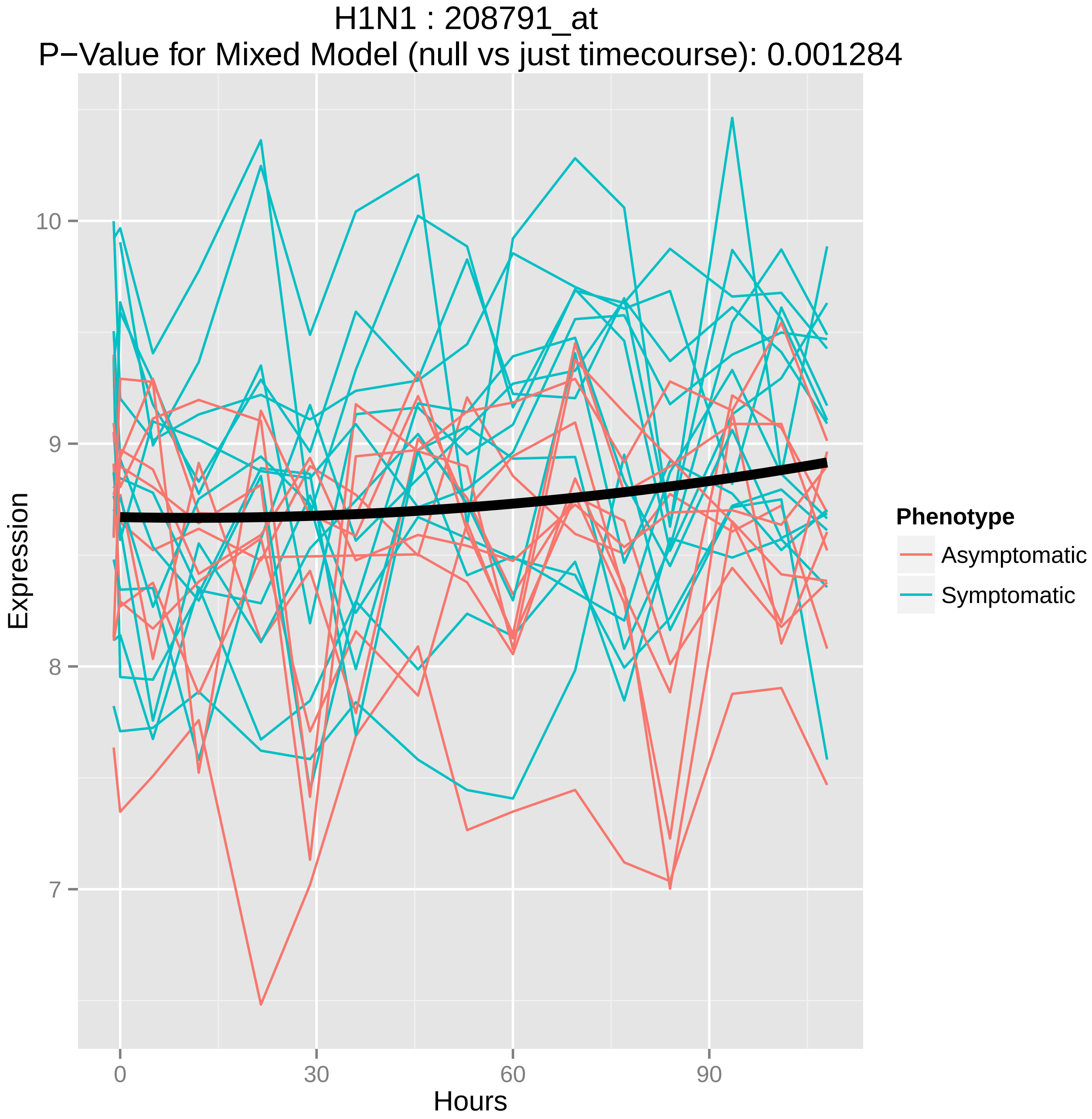
H1N1 : 208791_at
P−Value for Mixed Model (null vs just timecourse): 0.001284
Expression
10
9
8
7
0
30
60
90
Hours
Phenotype
Asymptomatic
Symptomatic

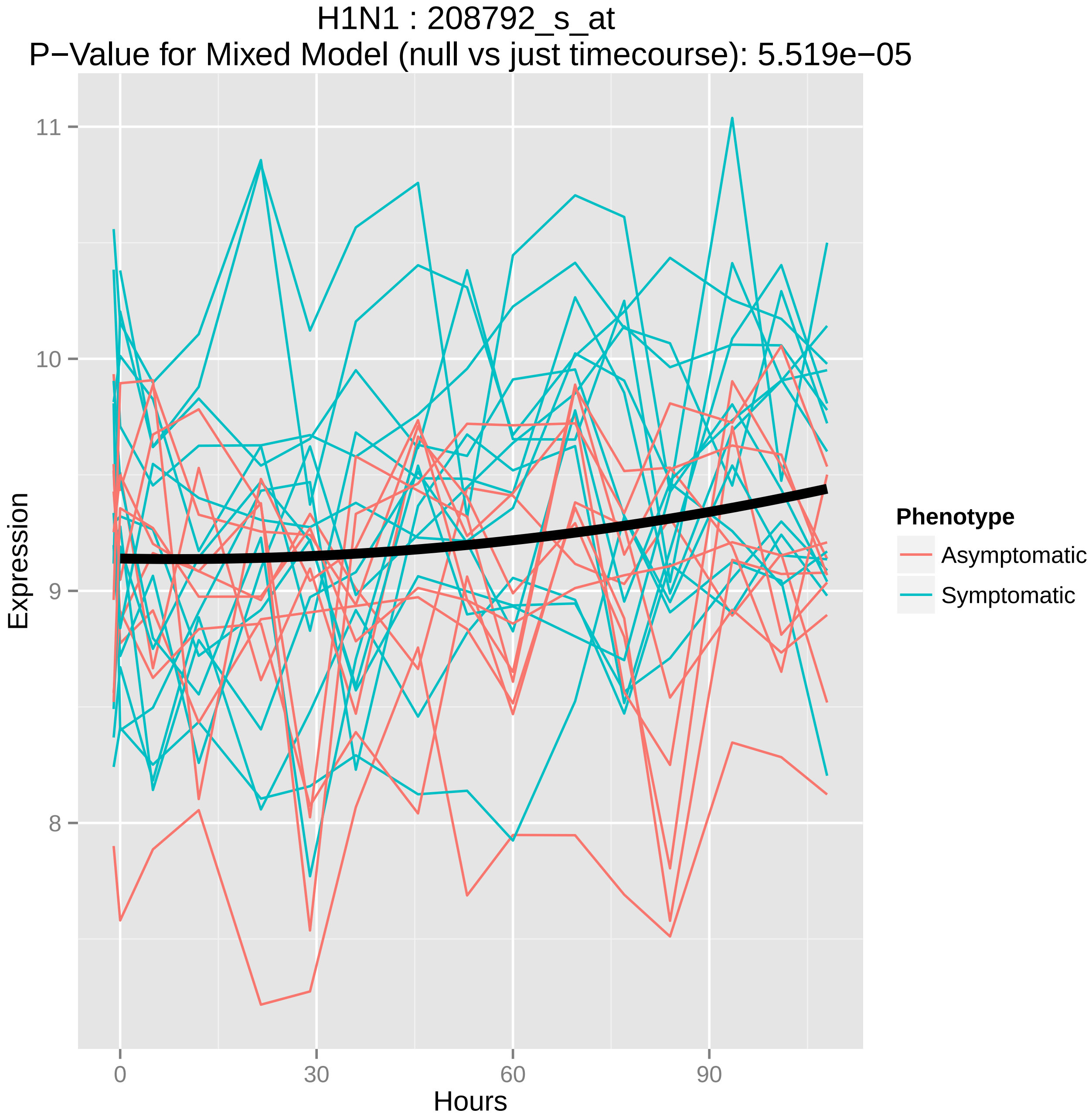

H1N1 : 208792_s_at
P−Value for Mixed Model (null vs just timecourse): 5.519e−05
11
10
9
8
Expression
0
30
60
90
Hours
Phenotype
Asymptomatic
Symptomatic

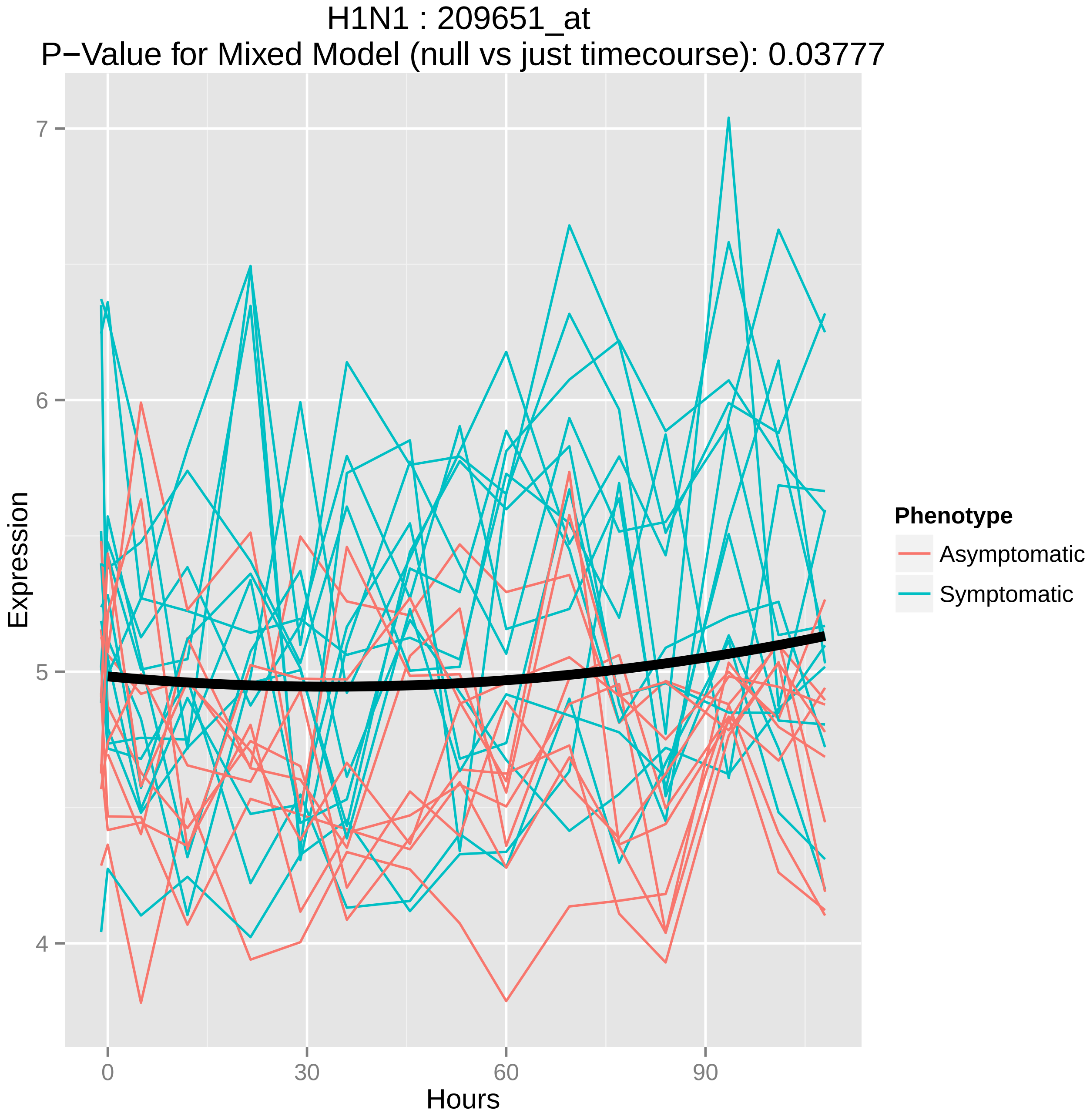
H1N1 : 209651_at
P−Value for Mixed Model (null vs just timecourse): 0.03777
7
6
5
4
Expression
0
30
60
90
Hours
Phenotype
Asymptomatic
Symptomatic

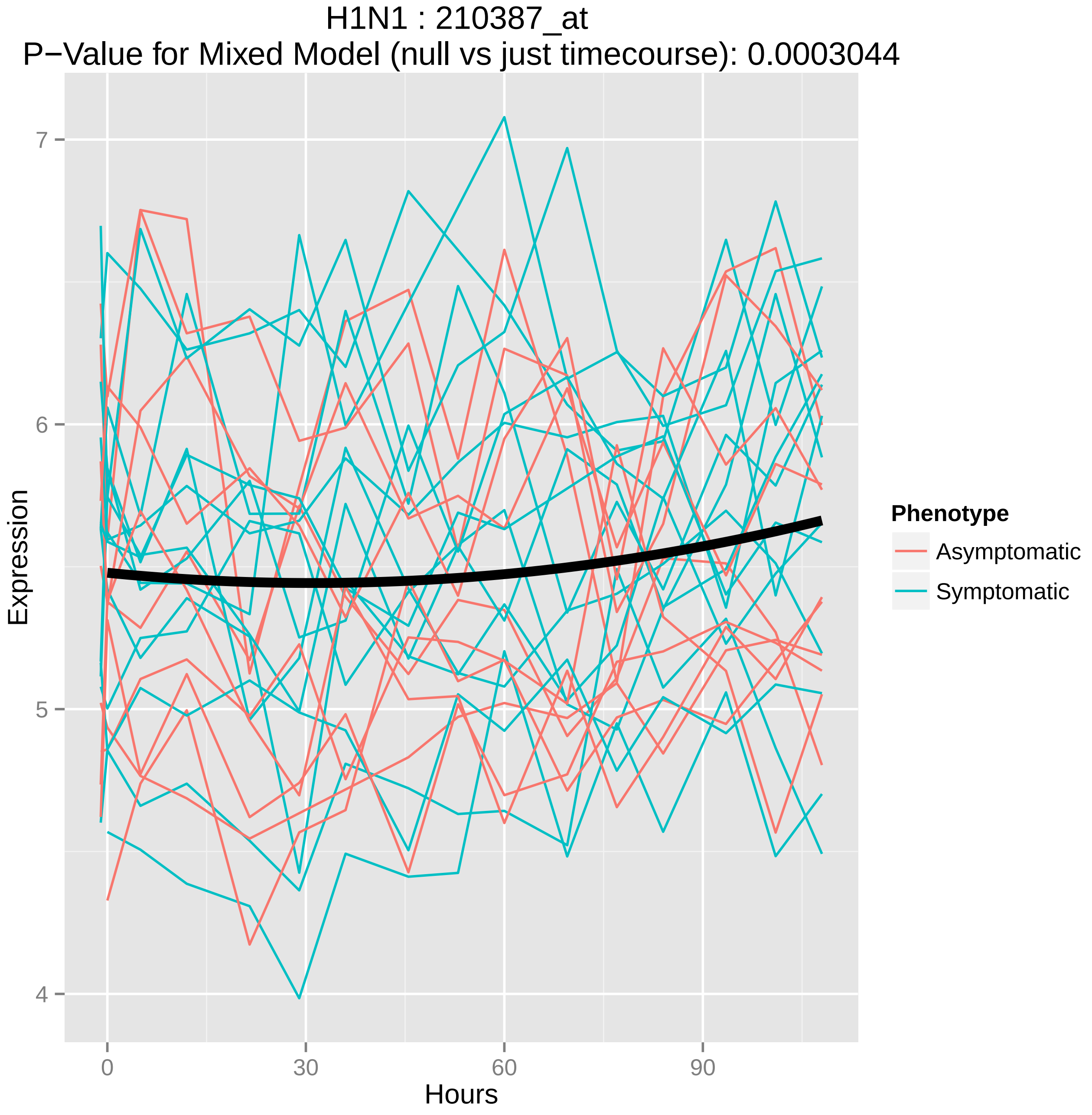

H1N1 : 210387_at
P−Value for Mixed Model (null vs just timecourse): 0.0003044
Expression
Hours
7
6
5
4
0
30
60
90
Phenotype
Asymptomatic
Symptomatic

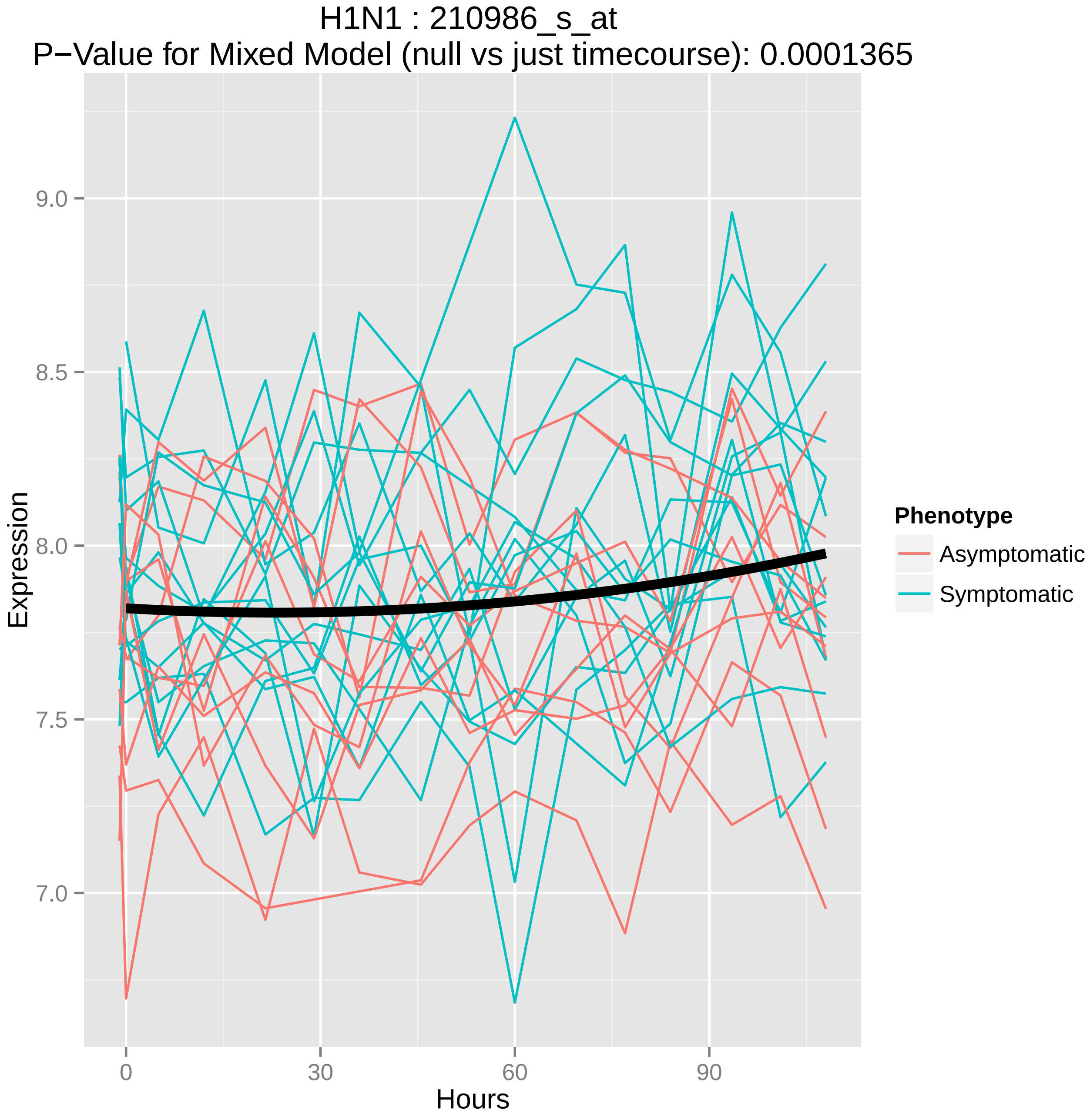
H1N1 : 210986_s_at
P−Value for Mixed Model (null vs just timecourse): 0.0001365
Expression
9.0
8.5
8.0
7.5
7.0
0
30
60
90
Hours
Phenotype
Asymptomatic
Symptomatic

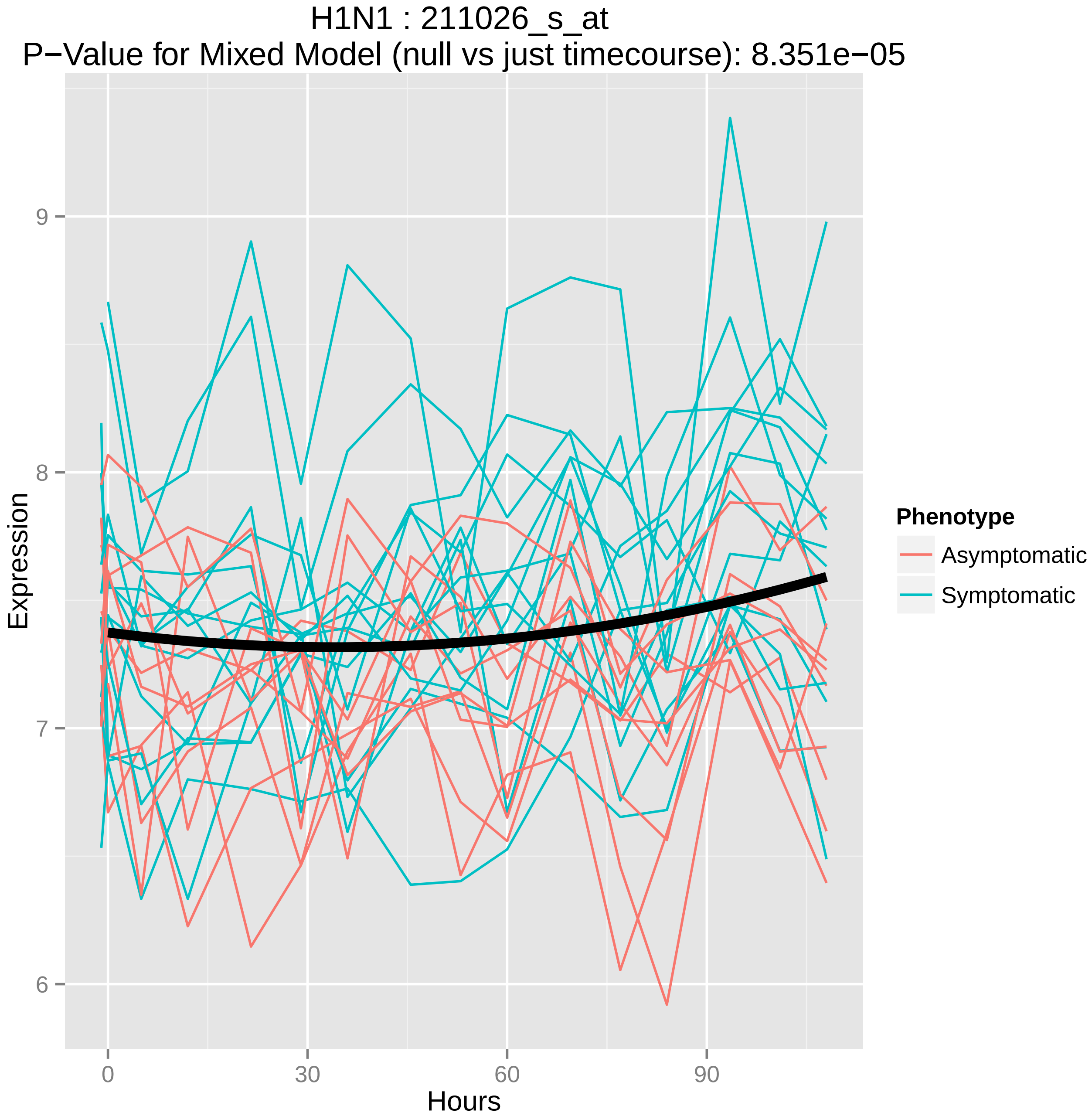
H1N1 : 211026_s_at
P−Value for Mixed Model (null vs just timecourse): 8.351e−05
Expression
9
8
7
6
0
30
60
90
Hours
Phenotype
Asymptomatic
Symptomatic

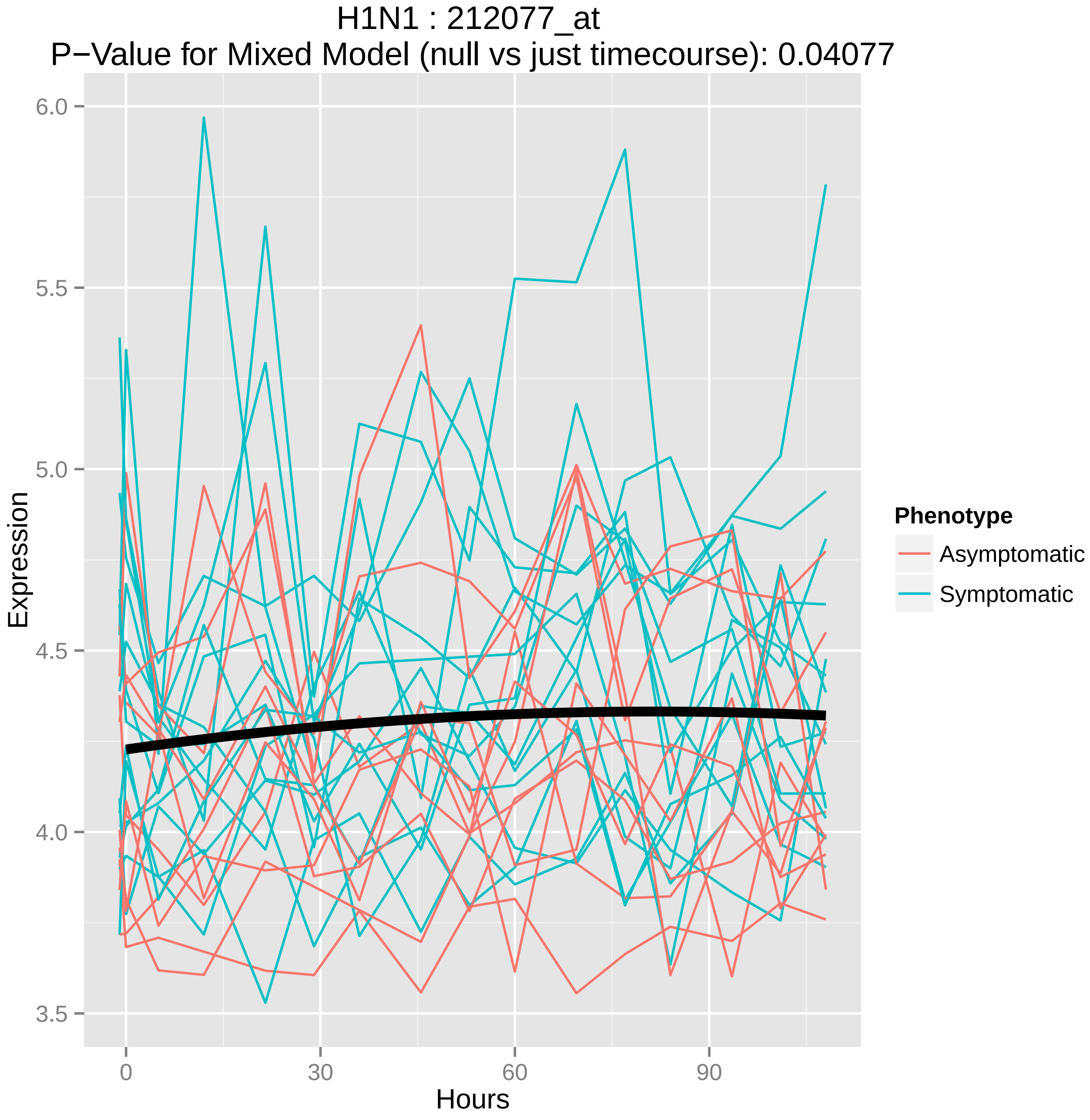
H1N1 : 212077_at
P−Value for Mixed Model (null vs just timecourse): 0.04077
Expression
6.0
5.5
5.0
4.5
4.0
3.5
0
30
60
90
Hours
Phenotype
Asymptomatic
Symptomatic

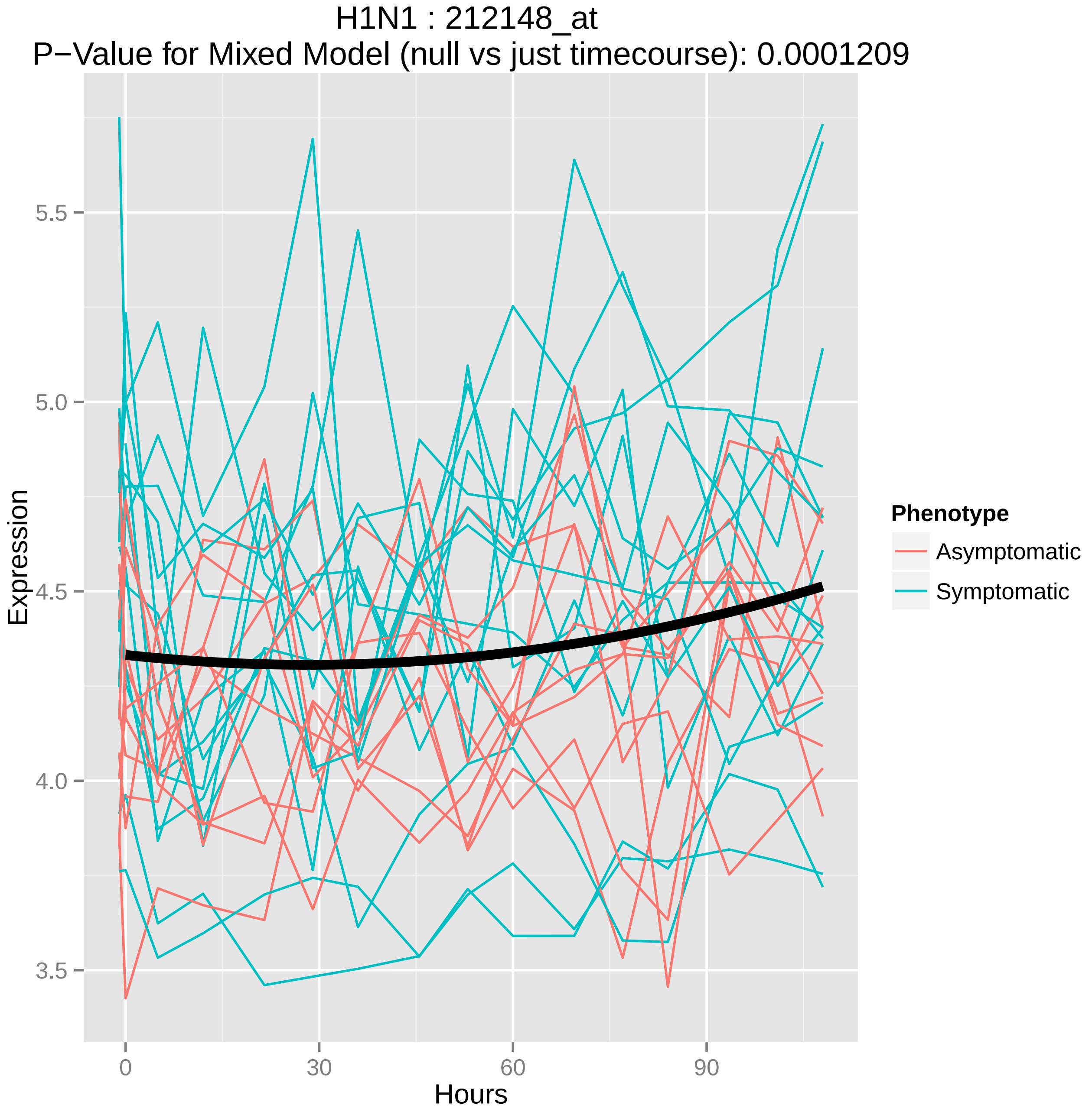
H1N1 : 212148_at
P−Value for Mixed Model (null vs just timecourse): 0.0001209
Expression
5.5
5.0
4.5
4.0
3.5
0
30
60
90
Hours
Phenotype
Asymptomatic
Symptomatic

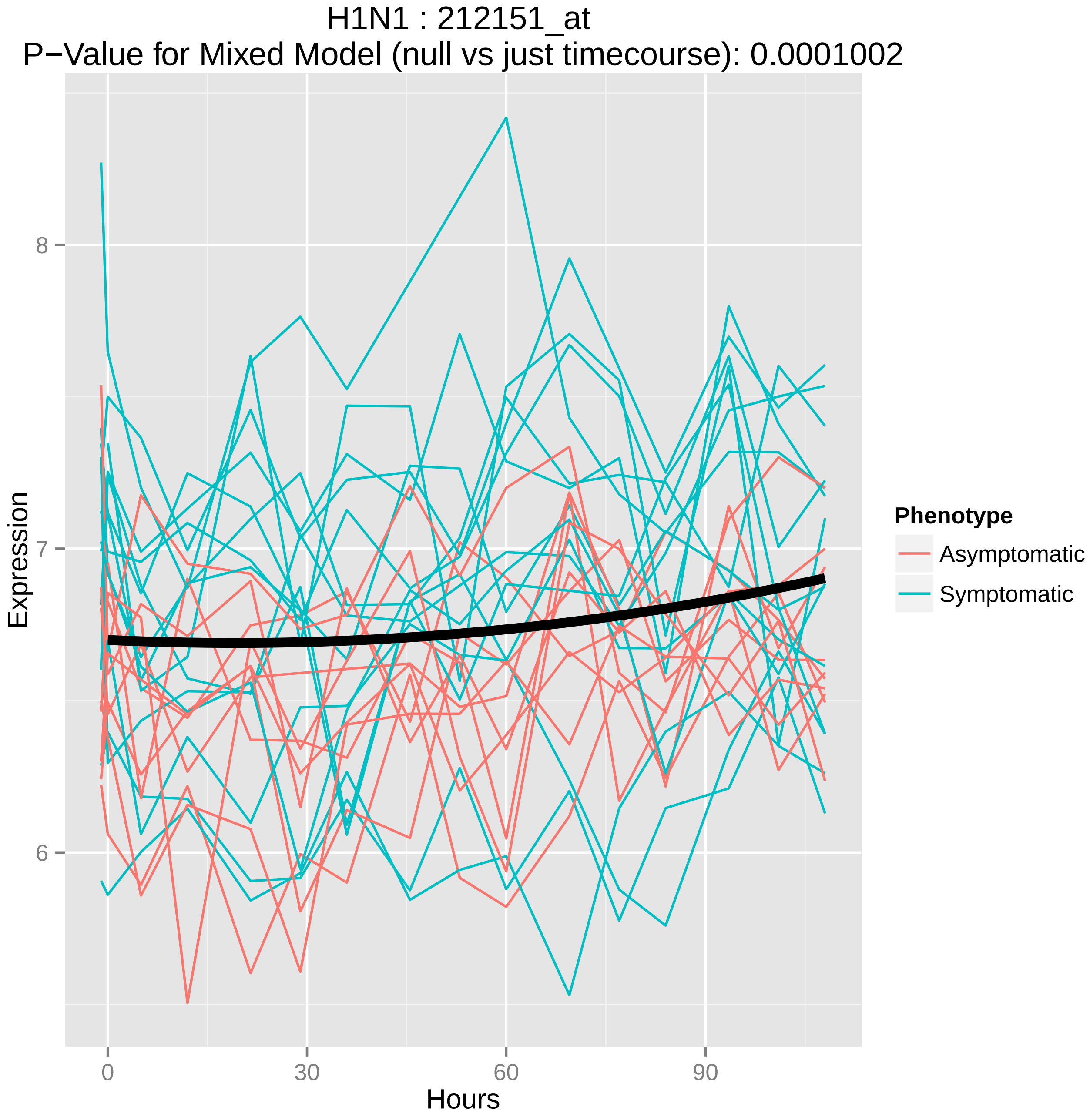

H1N1 : 212151_at
P−Value for Mixed Model (null vs just timecourse): 0.0001002
Expression
8
7
6
0
30
60
90
Hours
Phenotype
Asymptomatic
Symptomatic

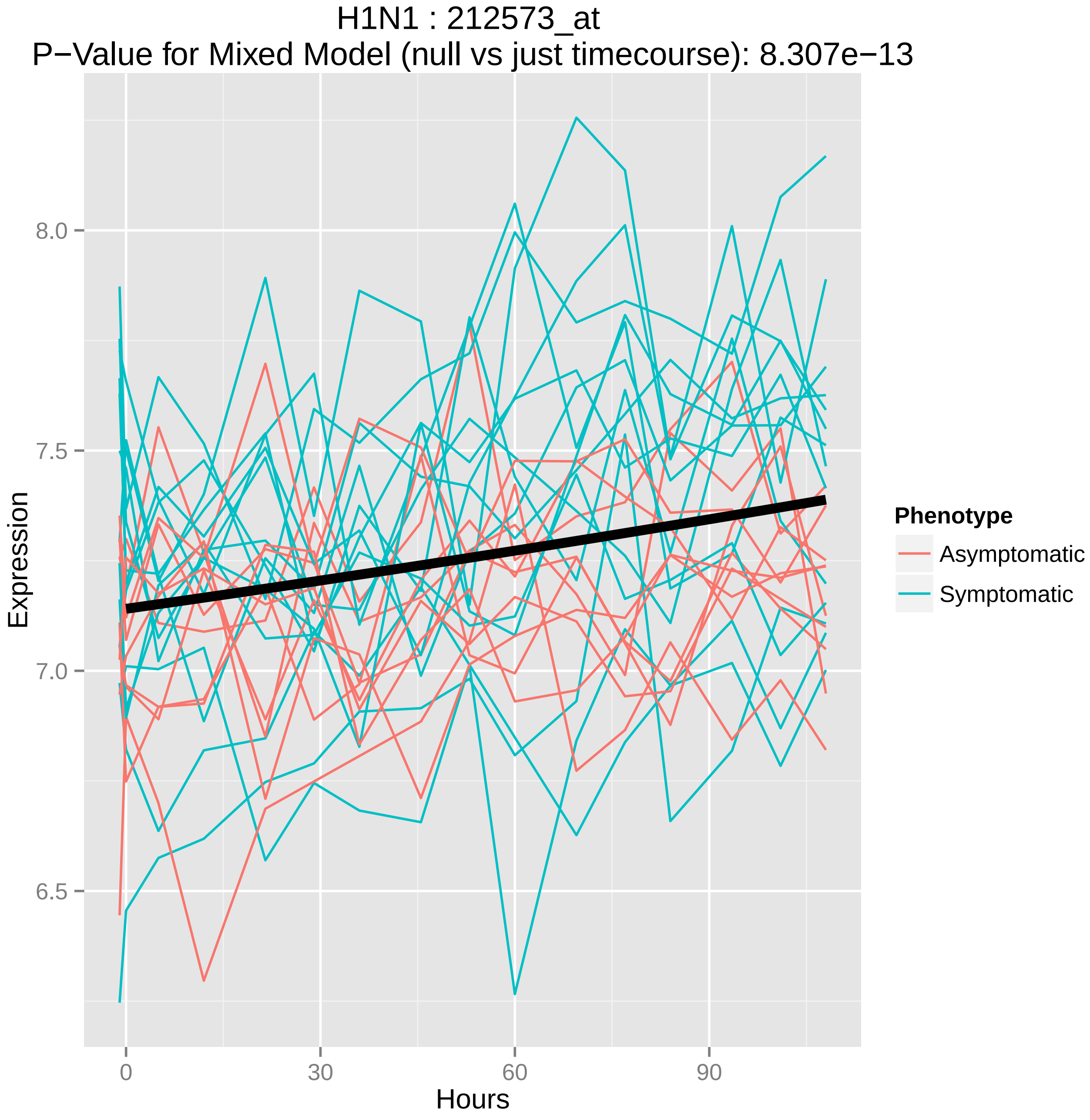
H1N1 : 212573_at
P−Value for Mixed Model (null vs just timecourse): 8.307e−13
Expression
8.0
7.5
7.0
6.5
0
30
60
90
Hours
Phenotype
Asymptomatic
Symptomatic

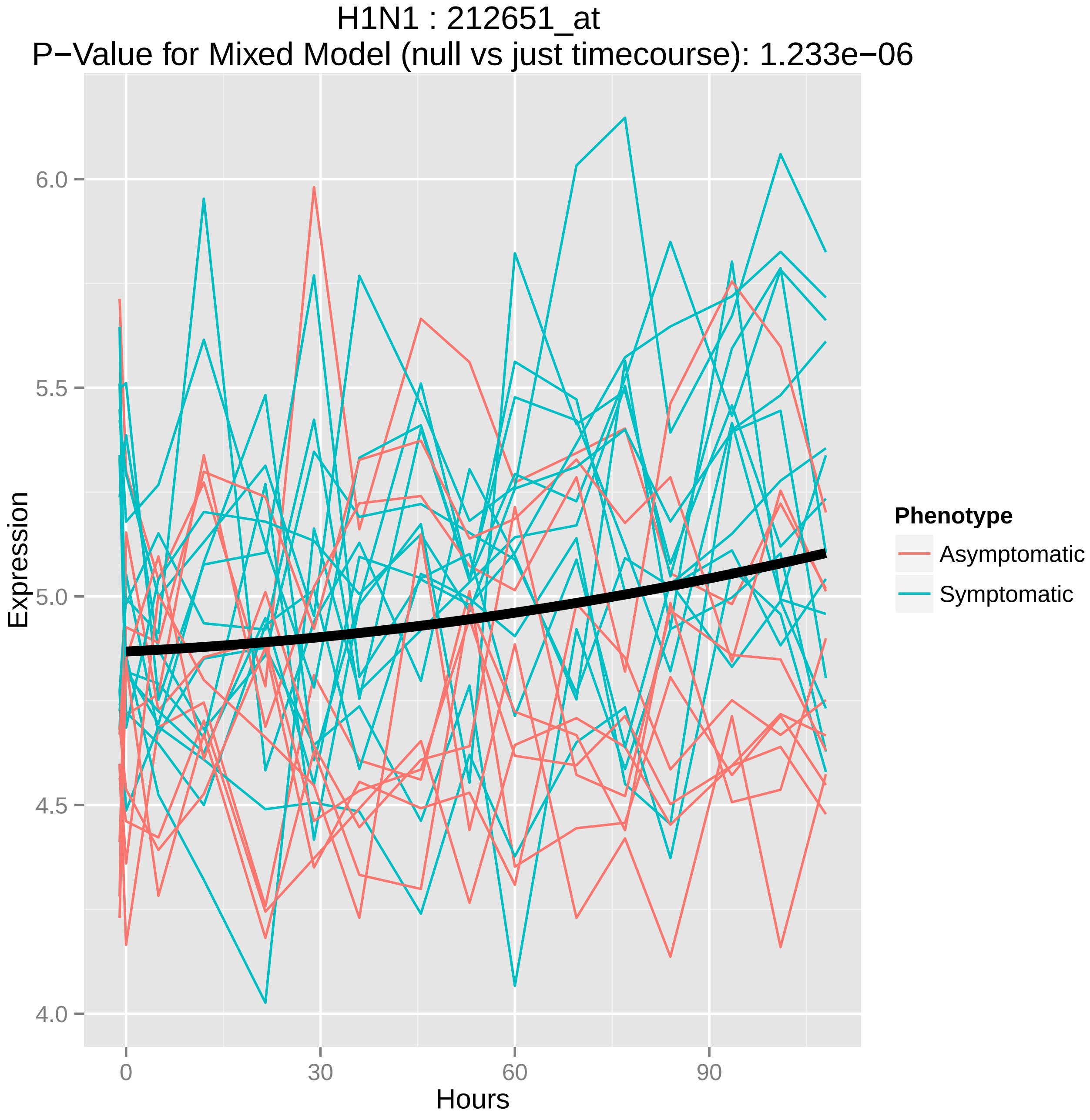
H1N1 : 212651_at
P−Value for Mixed Model (null vs just timecourse): 1.233e−06
Expression
6.0
5.5
5.0
4.5
4.0
0
30
60
90
Hours
Phenotype
Asymptomatic
Symptomatic

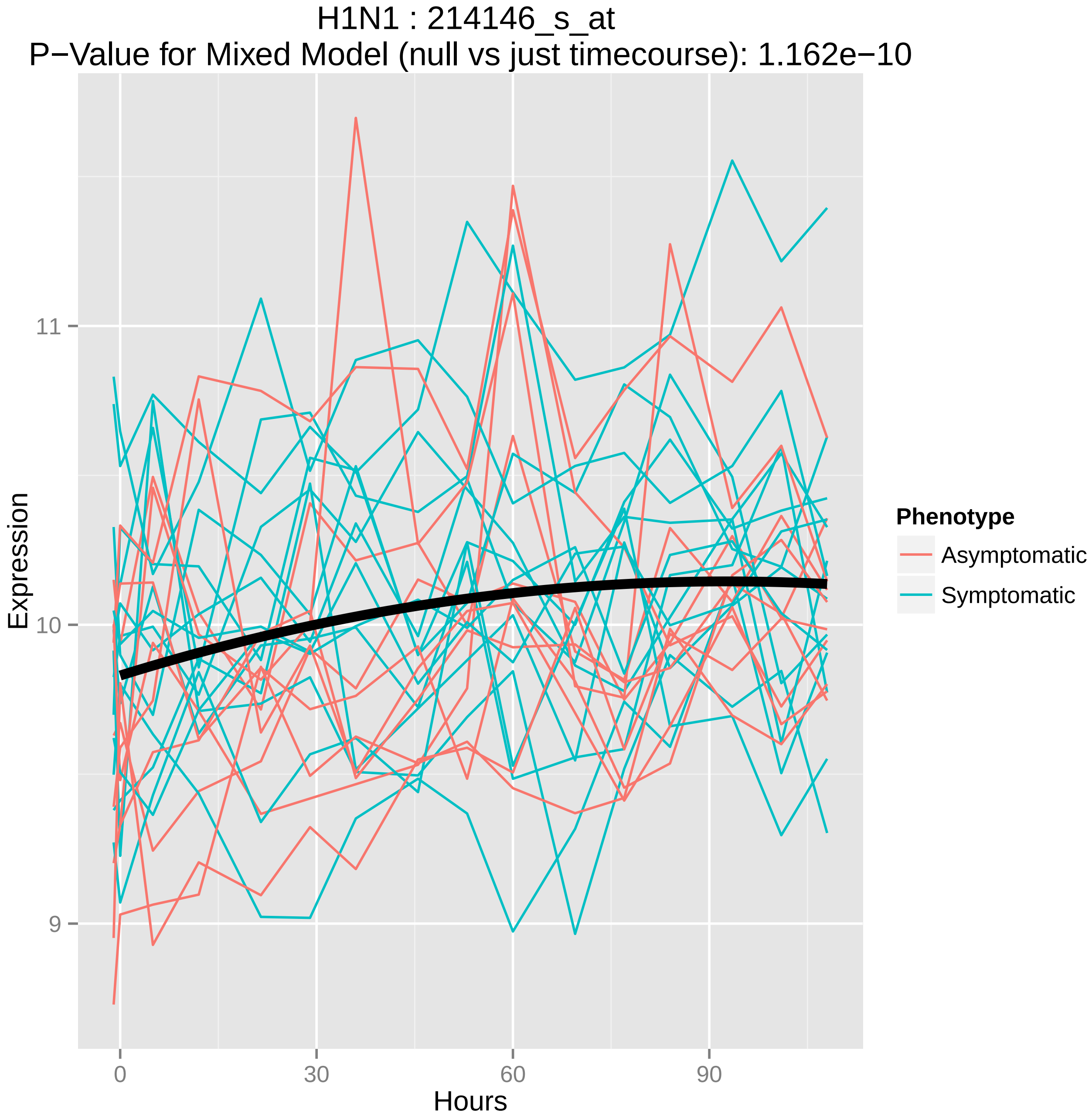

H1N1 : 214146_s_at
P−Value for Mixed Model (null vs just timecourse): 1.162e−10
Expression
11
10
9
0
30
60
90
Hours
Phenotype
Asymptomatic
Symptomatic

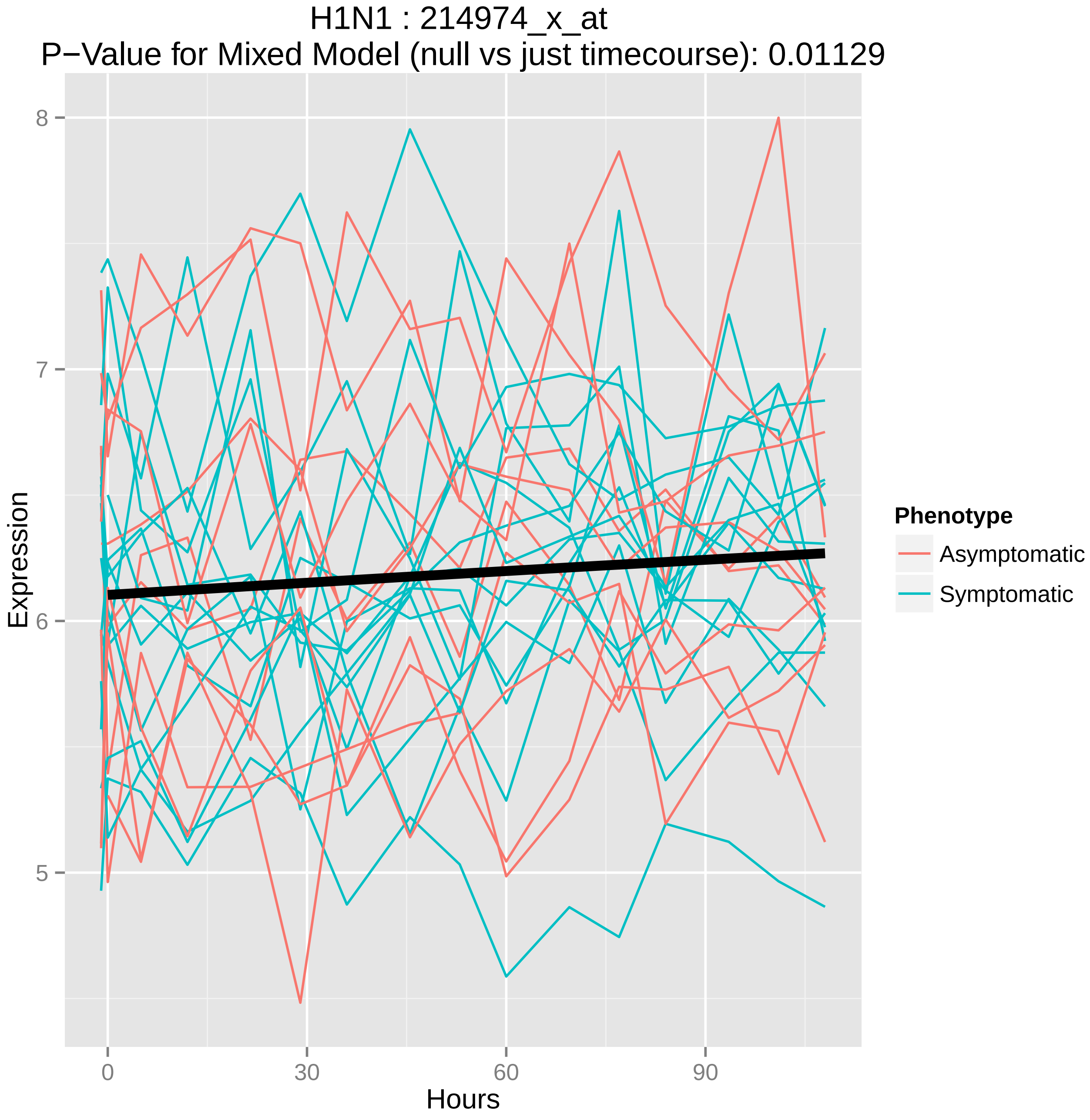
H1N1 : 214974_x_at
P−Value for Mixed Model (null vs just timecourse): 0.01129
Expression
8
7
6
5
0
30
60
90
Hours
Phenotype
Asymptomatic
Symptomatic

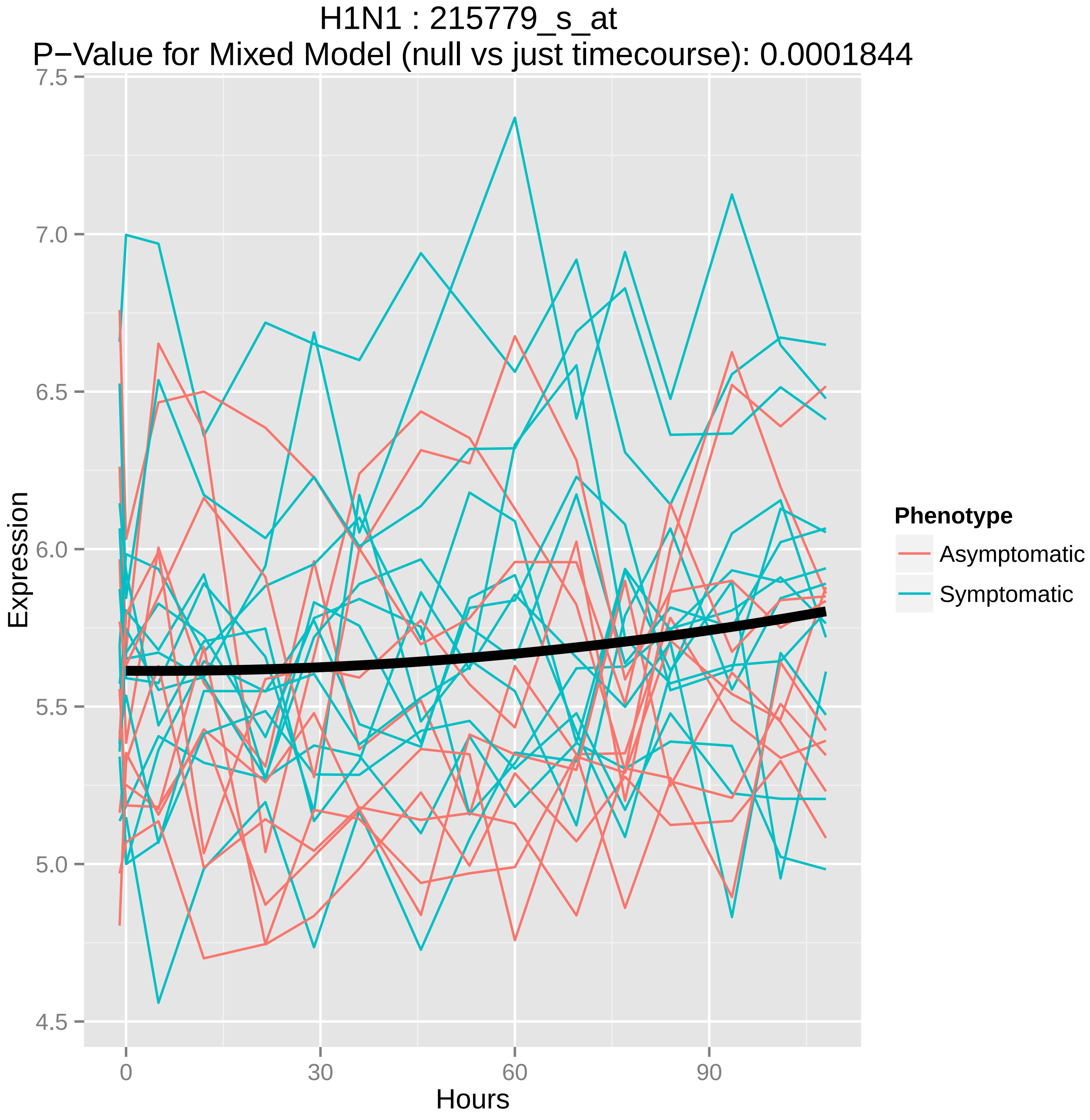
H1N1 : 215779_s_at
P−Value for Mixed Model (null vs just timecourse): 0.0001844
Expression
Hours
7.5
7.0
6.5
6.0
5.5
5.0
4.5
0
30
60
90
Phenotype
Asymptomatic
Symptomatic

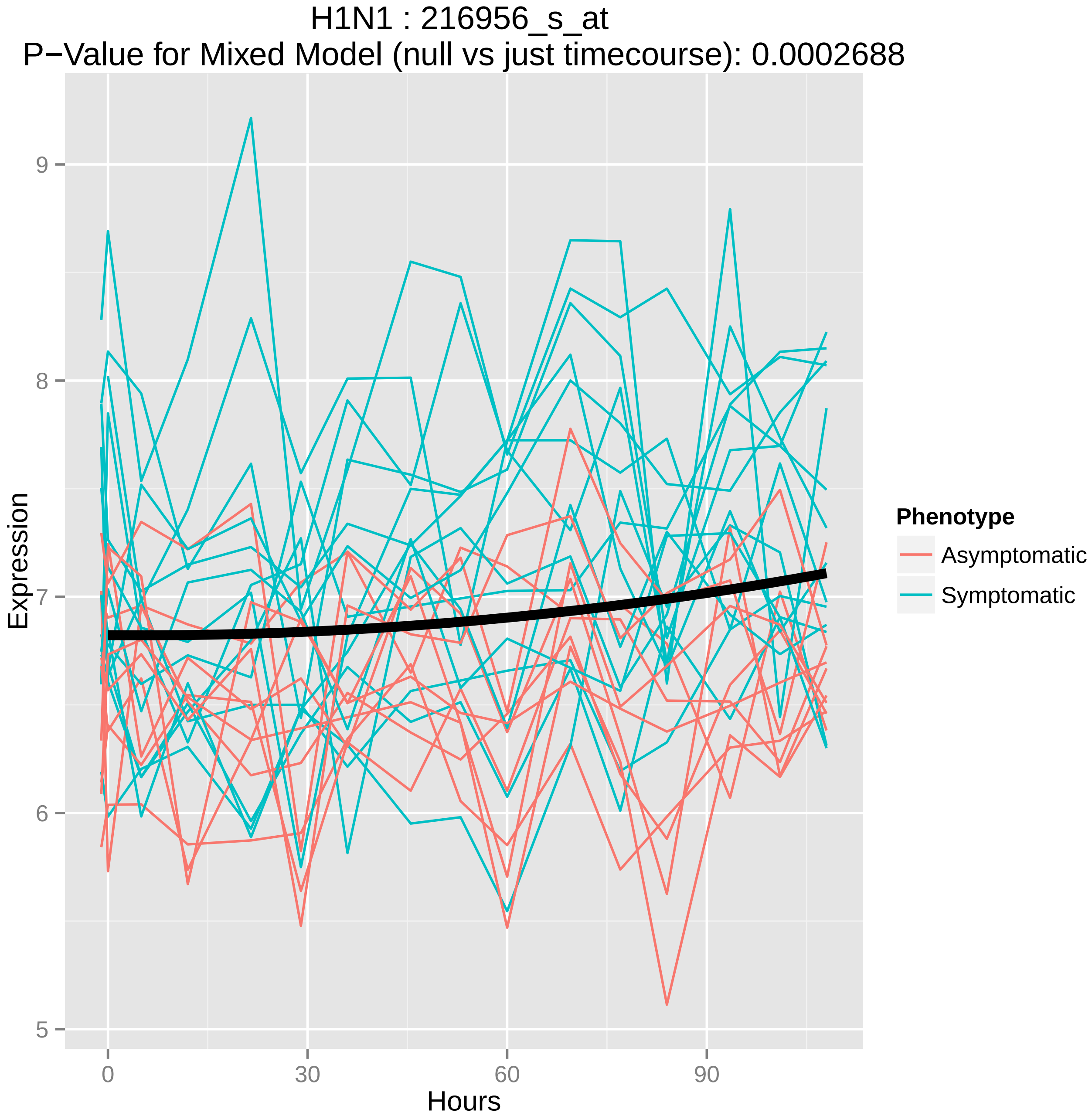
H1N1 : 216956_s_at
P−Value for Mixed Model (null vs just timecourse): 0.0002688
Expression
Hours
9
8
7
6
5
0
30
60
90
Phenotype
Asymptomatic
Symptomatic

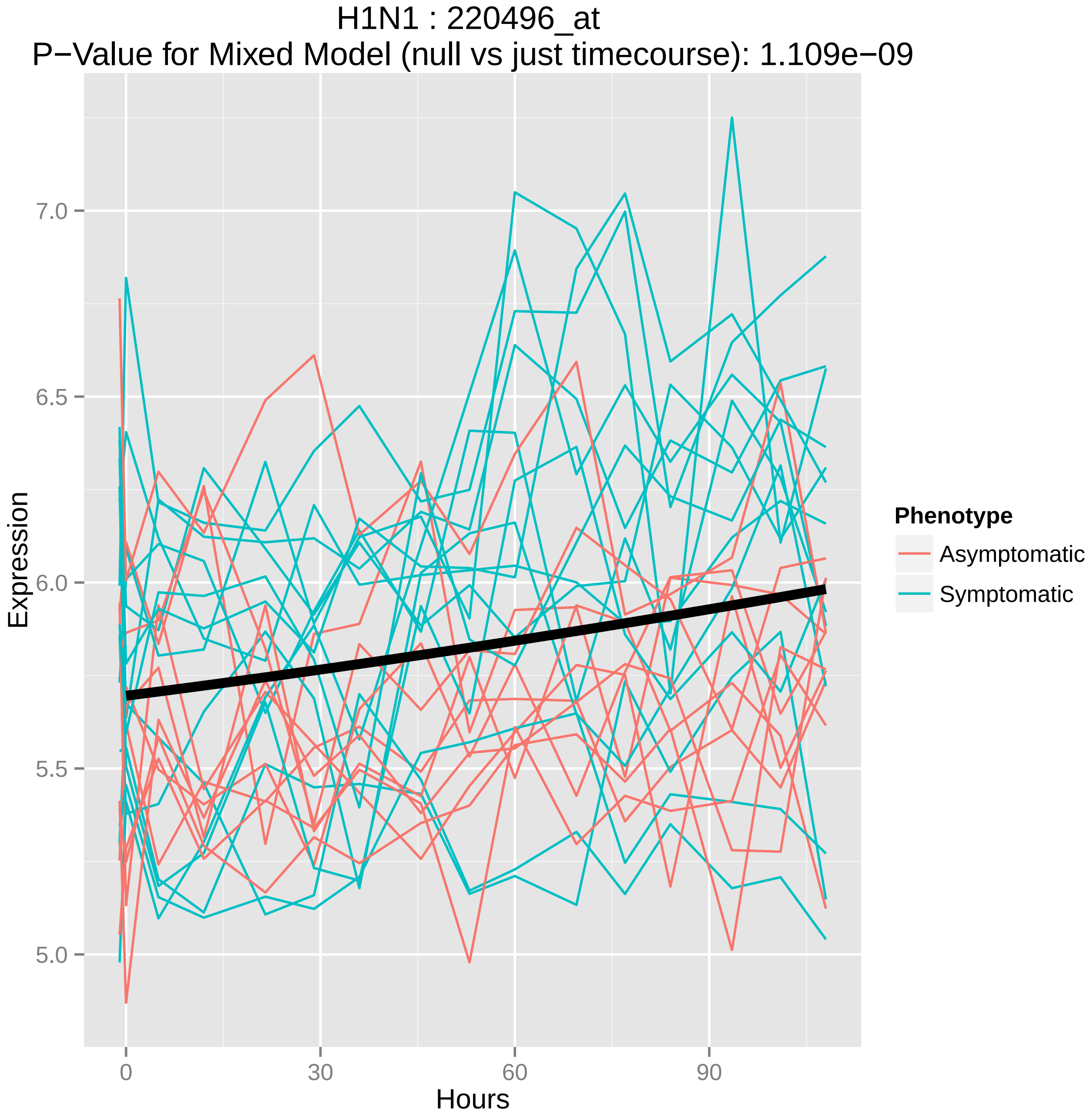

H1N1 : 220496_at
P−Value for Mixed Model (null vs just timecourse): 1.109e−09
Expression
7.0
6.5
6.0
5.5
5.0
0
30
60
90
Hours
Phenotype
Asymptomatic
Symptomatic
