# Supplementary material for: Gene Expression Profiles Link Respiratory Viral Infection, Platelet Response to Aspirin, and Acute Myocardial Infarction
Source: PLoS One. 2015 Jul 20;10(7):e0132259. doi: 10.1371/journal.pone.0132259 (PMC4507878; doi:10.1371/journal.pone.0132259)
Supplement: S1 Table — (PDF) [file pone.0132259.s002.pdf]

S1 Table. Numbers subjects with available RNA data at each time point after viral exposure in each viral cohort.

Time (hours)	H1N1	H3N2	HRV	RSV
0 (baseline)	24	32	38	37
4	0	0	19	0
5	24	17	0	8
8	0	0	16	0
12	24	17	20	19
16	0	0	20	0
20	0	0	20	0
21.5	24	15	0	8
24	0	0	18	0
29	23	15	0	18
30	0	0	20	0
36	23	15	18	8
42	0	0	19	0
45.5	23	15	0	8
48	0	0	19	0
53	22	16	0	8
60	24	15	0	8
69.5	23	17	0	8
72	0	0	20	0
77	24	16	0	8
84	24	15	0	8
93.5	24	17	0	8
96	0	0	20	0
101	24	17	0	8
108	24	15	0	8
118	0	0	0	17
125	0	0	0	7
132	0	0	0	7
142	0	0	0	20
166	0	0	0	8
